# Ring-size-selective construction of fluorine-containing carbocycles via intramolecular iodoarylation of 1,1-difluoro-1-alkenes

**DOI:** 10.3762/bjoc.13.266

**Published:** 2017-12-14

**Authors:** Takeshi Fujita, Ryo Kinoshita, Tsuyoshi Takanohashi, Naoto Suzuki, Junji Ichikawa

**Affiliations:** 1 Division of Chemistry, Faculty of Pure and Applied Sciences, University of Tsukuba, Tsukuba, Ibaraki 305-8571, Japan

**Keywords:** alkenes, carbocycles, cyclization, electrophilic activation, fluorine, iodine

## Abstract

1,1-Difluoro-1-alkenes bearing a biaryl-2-yl group effectively underwent site-selective intramolecular iodoarylation by the appropriate cationic iodine species. Iodoarylation of 2-(2-aryl-3,3-difluoroallyl)biaryls proceeded via regioselective carbon–carbon bond formation at the carbon atoms in β-position to the fluorine substituents, thereby constructing dibenzo-fused six-membered carbocycles bearing a difluoroiodomethyl group. In contrast, 2-(3,3-difluoroallyl)biaryls underwent a similar cyclization at the α-carbon atoms to afford ring-difluorinated seven-membered carbocycles.

## Introduction

As 1,1-difluoro-1-alkenes have an electron-deficient carbon–carbon double bond, they readily undergo intramolecular substitution of nucleophiles through an addition–elimination mechanism [1–2]. Thus, under basic conditions, they serve as useful precursors for ring-fluorinated heterocycles and carbocycles that are promising candidates for pharmaceuticals, agrochemicals, and functional materials. In contrast, the cationic cyclization of 1,1-difluoro-1-alkenes using electrophilic reagents (under acidic conditions) has been quite limited because of their low electron densities caused by fluorine substituents [3–5]. Despite the limitation, the cationic cyclization of difluoroalkenes possesses high potential for the synthesis of fluorine-containing cyclic compounds. Thus, the development of this type of cyclization is highly desirable to further expand the utility of difluoroalkenes in organic synthesis.

We have already achieved the metal-catalyzed and acid-mediated cationic cyclization of 1,1-difluoro-1-alkenes. In the former case, we reported the palladium [6–10], indium [10–13], and silver-catalyzed construction of ring-fluorinated carbocycles and heterocycles [14]. In the latter case, the domino-Friedel–Crafts-type cyclization proceeded via the cleavage of two carbon–fluorine bonds to afford polycyclic aromatic hydrocarbons [15–21]. Both types of cationic cyclization proceeded exclusively at the carbon atoms α to the fluorine substituents, because the β-selective metalation or protonation of difluoroalkenes generates α-fluorocarbocations, which were stabilized by the resonance effect of fluorine substituents.

**Scheme 1:**
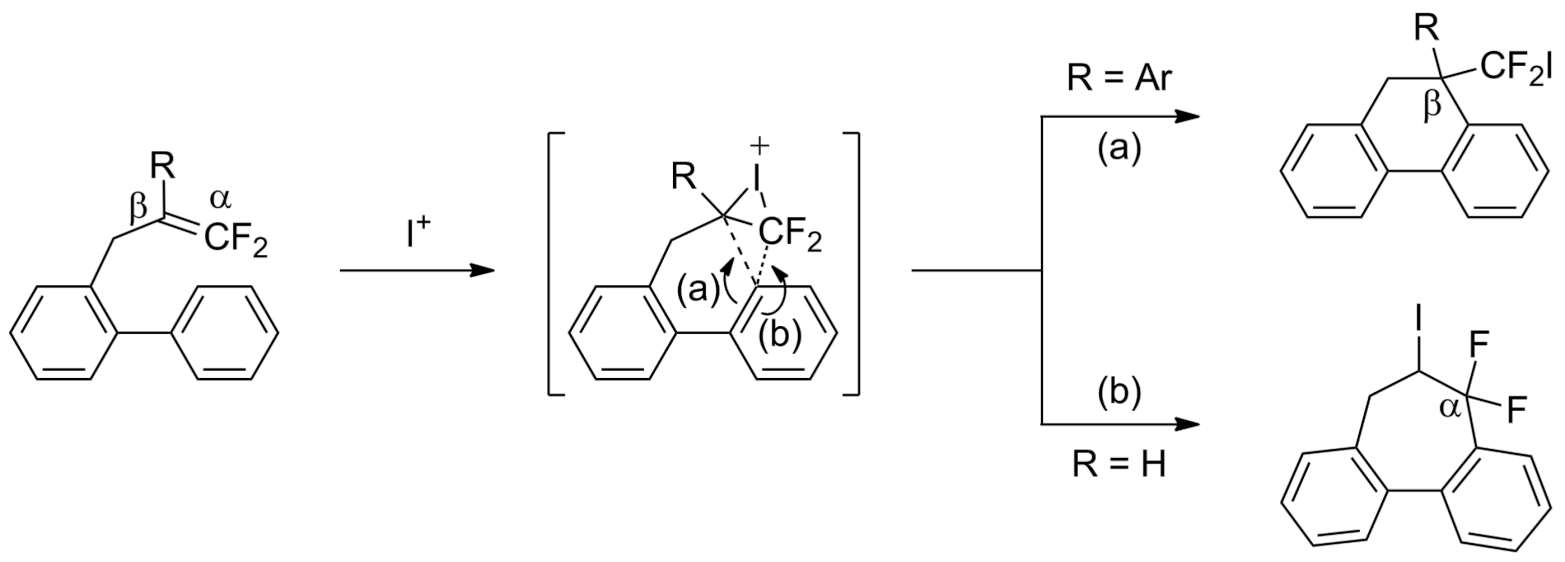
Intramolecular site-selective iodoarylation of 1,1-difluoro-1-alkenes bearing a biaryl group.

In the course of our studies on the cationic cyclization of 1,1-difluoro-1-alkenes, we undertook an investigation of their iodine-mediated cyclization. Three-membered iodonium intermediates generated in the reaction course were expected to exhibit switchable regioselectivities [22]. This is because their cationic charge might be less localized on the carbon atoms α to the fluorine substituents, as compared to the aforementioned cationic intermediates [23–27]. Thus, we examined and eventually achieved complete control over the regioselectivity at the carbon atoms in β-position as well as those in α-position to the fluorine in the intramolecular Friedel–Crafts-type iodoarylation of 1,1-difluoro-1-alkenes bearing a biaryl group. Among the 1,1-difluoro-1-alkenes examined, 2-(2-aryl-3,3-difluoroallyl)biaryls underwent cyclization at the carbon atoms in β-position to the fluorine substituents to construct six-membered carbocycles bearing a difluoroiodomethyl group (Scheme 1a). On the other hand the cyclization of 2-(3,3-difluoroallyl)biaryls proceeded at the α-carbon atoms to give ring-fluorinated seven-membered carbocycles (Scheme 1b) [28].

## Results and Discussion

**Scheme 2:**
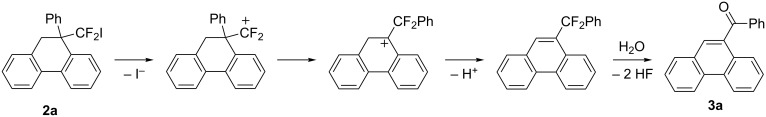
Mechanism for formation of **3a**.

First, we sought an electrophilic iodine species suitable for the intramolecular iodoarylation of 2-(2-aryl-3,3-difluoroallyl)biaryls **1** using 2-(2-phenyl-3,3-difluoroallyl)biphenyl (**1a**) as a model substrate. To generate a highly reactive, cationic iodine species, several iodine sources were used with acid or metal activators (Table 1, entries 1–3). Upon treatment with *N*-iodosuccinimide (NIS) and trimethylsilyl trifluoromethanesulfonate (TMSOTf) in a 1:1 mixed solvent of 1,1,1,3,3,3-hexafluoropropan-2-ol (HFIP) and dichloromethane, **1a** afforded the expected iodoarylation product **2a** and its overreacted product, 9-benzoylphenanthrene (**3a**), in 34% and 15% yields, respectively (Table 1, entry 1). Ketone **3a** was formed probably via the sequence consisting of iodide elimination from **2a**, 1,2-migration of the phenyl group, and deprotonation, followed by hydrolysis of the resulting doubly activated benzylic difluoromethylene unit (Scheme 2). Neither a combination of bis(pyridine)iodonium (IPy_2_BF_4_) and trifluoromethanesulfonic acid nor a combination of I_2_ and silver(I) triflate improved the yield of **2a** (Table 1, entries 2 and 3). Although iodine monochloride, which is known as a cationic iodine species, afforded only ketone **3a** (Table 1, entry 4), its pyridine complex (PyICl) exclusively afforded the iodoarylation product **2a** in 31% yield (Table 1, entry 5). The use of a 9:1 mixed solvent of HFIP and CH_2_Cl_2_ improved the yield of **2a** to 85% (Table 1, entry 6). Lastly, increasing the concentration up to 0.075 M suppressed the formation of **3a** and selectively afforded **2a** in 91% yield (Table 1, entry 7). In this reaction, the nucleophilic benzene ring attacked the carbon atom β to the fluorine substituents of the cyclic iodonium intermediate, which was derived from **1a** and PyICl. This indicates that the cationic charge in the cyclic iodonium intermediate might be localized at the β-carbon atom because of stabilization by the proximal phenyl group [28].

**Table 1:** Screening of conditions for the iodoarylation of **1a**.

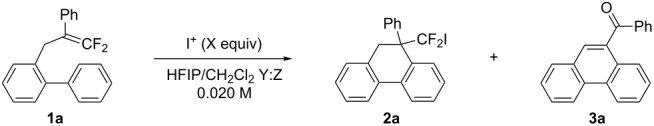					

entry	I^+^ (X equiv)	Y:Z	conditions	**2a** (%)^a^	**3a** (%)^a^

1	NIS (1.2), TMSOTf (1.2)	1:1	0 °C, 40 min	34	15
2	IPy_2_BF_4_ (1.0), TfOH (2.0)	1:1	0 °C, 1.5 h	N.D.^b^	N.D.^b^
3	I_2_ (1.2), AgOTf (1.2)	1:1	0 °C, 1.5 h	16	33
4	ICl (2.0)	1:1	0 °C, 20 min	N.D.^b^	43
5	PyICl (2.0)	1:1	0 °C, 1 h	31	N.D.^b^
6	PyICl (2.0)	9:1	0 °C, 1 h	85	12
7^c^	PyICl (2.0)	9:1	0 °C, 1 h	91	1

^a^Yield was determined by ^19^F NMR spectroscopy using PhCF_3_ as an internal standard. ^b^N.D. = not detected. ^c^0.075 M.

**Figure 1:**
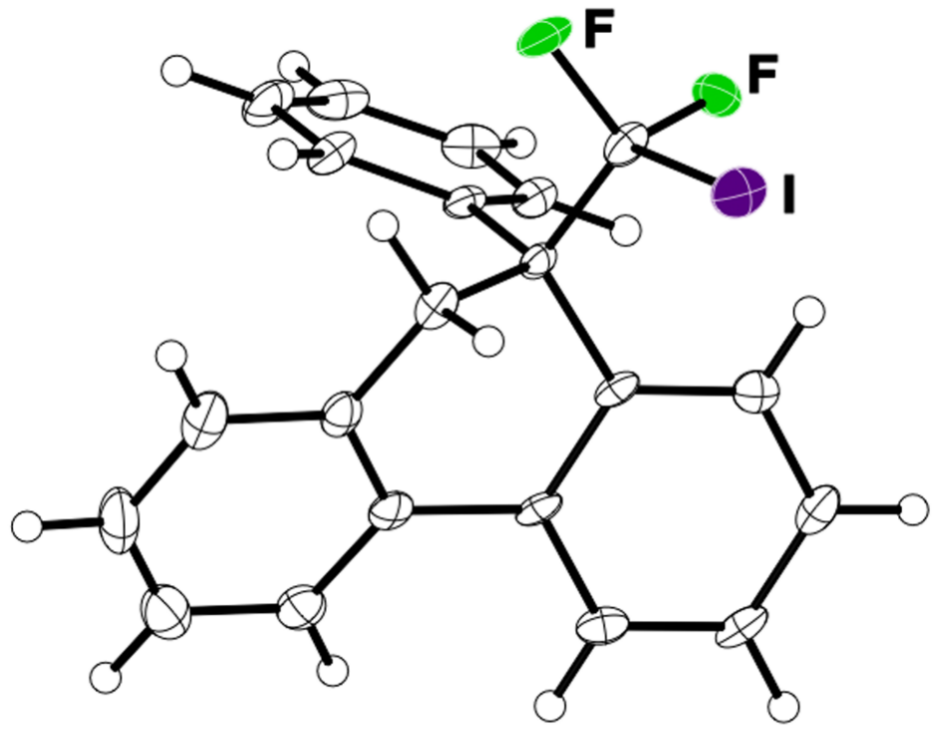
ORTEP diagram of **2a** with 50% ellipsoid probability.

Under the optimal conditions obtained above, iodoarylation of several 2-(2-aryl-3,3-difluoroallyl)biaryls **1** was examined (Table 2). Difluoroiodomethylated dihydrophenanthrenes, **2a** and **2b**, bearing a phenyl and a biphenyl-4-yl group were obtained in 79% and 74% isolated yields, respectively. 2-(2-Aryl-3,3-difluoroallyl)biaryls **1c**–**e** bearing electron-donating substituents (4-Me, 3-Me, and 4-MeO) on the benzene ring attached to the vinylic position successfully underwent iodoarylation to afford the corresponding dihydrophenanthrenes **2c**–**e**. In contrast, electron-withdrawing substituents on similar positions hardly promoted the iodoarylation, which was presumably because of inefficient cyclic iodonium formation. Substrates **1f** and **1g** bearing electron-donating groups on the nucleophilic aryl groups also participated in the iodoarylation to afford the corresponding difluoroiodomethylated dihydrophenanthrenes **2f** and **2g** in 54% and 80% yields, respectively. However, with substrates bearing a strong electron-withdrawing group (e.g., CF_3_) on the nucleophilic benzene ring, the iodoarylation hardly proceeded. The unambiguous structural determination of the iodoarylation products **2** was accomplished by X-ray crystallographic analysis of **2a** (Figure 1), which revealed that the iodoarylation products **2** have six-membered carbocycles bearing a difluoroiodomethyl group.

**Table 2:** Construction of six-membered carbocycles via iodoarylation of **1**.

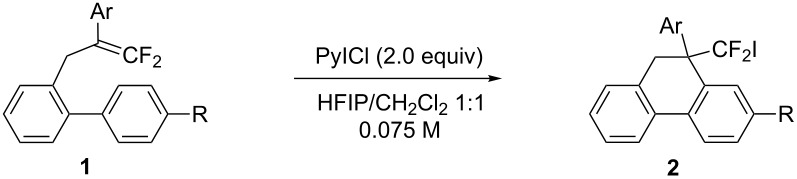				

entry	**1**	**2**	time	yield (%)^a^

1^b^	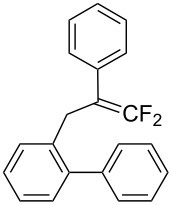 **1a**	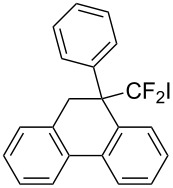 **2a**	1 h	79
2	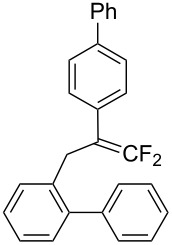 **1b**	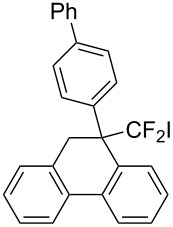 **2b**	2 h	74
3	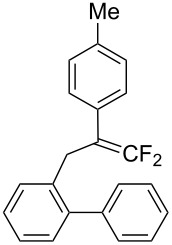 **1c**	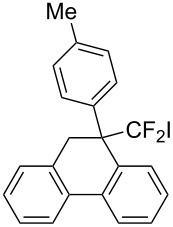 **2c**	35 min	82
4	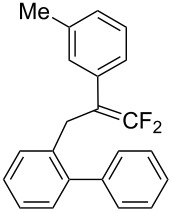 **1d**	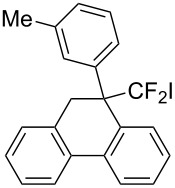 **2d**	1 h	53
5	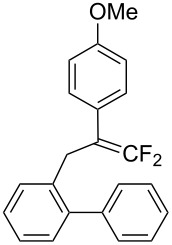 **1e**	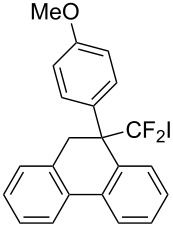 **2e**	25 min	83
6	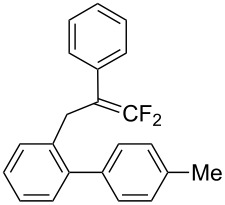 **1f**	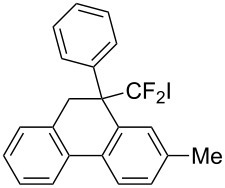 **2f**	1.5 h	54
7	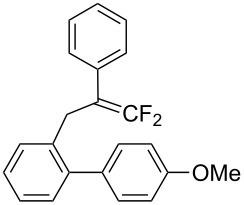 **1g**	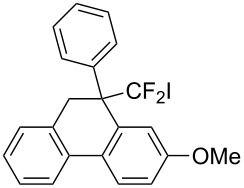 **2g**	1 h	80

^a^Isolated yield. ^b^HFIP/CH_2_Cl_2_ 9:1.

**Scheme 3:**
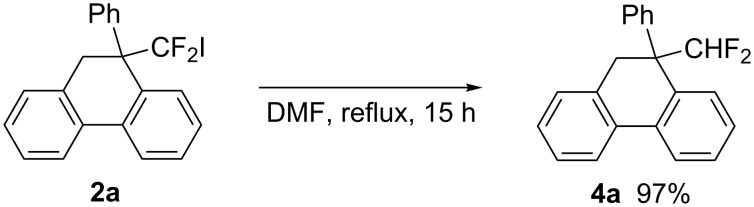
Transformation of a CF_2_I group of **2a** into a CHF_2_ group.

Further transformation of the difluoroiodomethyl group of **2a** was achieved by heating (Scheme 3). Thus, refluxing a DMF solution of **2a** for 15 h induced iodine–hydrogen exchange to afford difluoromethylated dihydrophenanthrene derivative **4a** in almost quantitative yield [29–30]. A difluoromethyl group functions as a hydrogen-bond donor and a bioisostere of a hydroxy group, as a result of which difluoromethyl-bearing compounds attract much attention as bioactive materials [31–32]. This sequence provides ready access to these compounds.

**Scheme 4:**
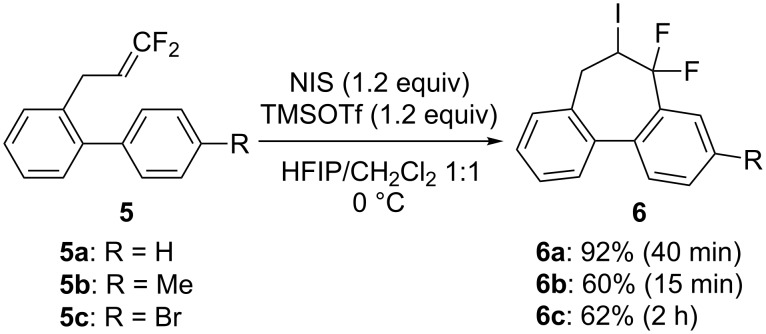
Construction of seven-membered carbocycles via iodoarylation of **5**.

**Figure 2:**
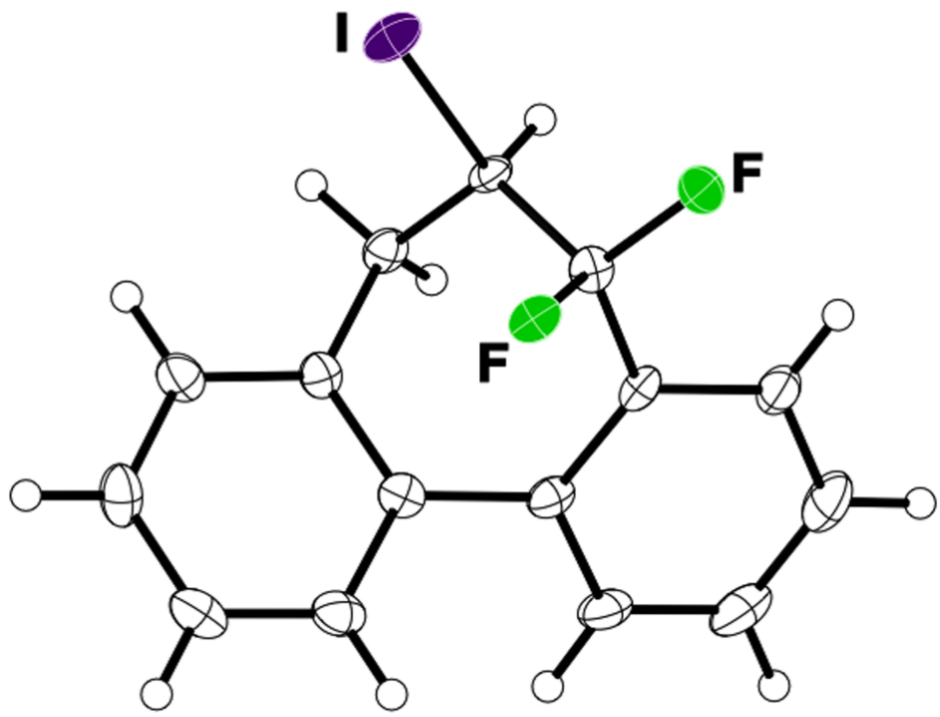
ORTEP diagram of **6a** with 50% ellipsoid probability.

Next, 2-(3,3-difluoroallyl)biphenyl (**5a**)**,** without an aryl group at its vinylic position, was subjected to the conditions examined in entries 1–3 of Table 1. In contrast to **1a**, iodoarylation of **5a** proceeded through C–C-bond formation exclusively at the carbon atoms α to the fluorine substituents to afford dibenzo-fused cycloheptane **6a** with geminal fluorine substituents on the ring in 92%, 38%, 49% yields, respectively. The thus-obtained selectivity might be attributed to the localization of the cationic charge at the carbon atoms α to the fluorine substituents in the three-membered iodonium intermediates. Since the combination of NIS and TMSOTf was found to be the best for an iodoarylation of **5**, the reactions of a couple of 2-(3,3-difluoroallyl)biaryls **5** were examined under the same conditions (Scheme 4). The iodoarylation of difluoroallylbiphenyl **5b**, bearing an electron-donating methyl group on the nucleophilic aryl group, was completed in 15 min to afford **6b** in 60% isolated yield. Brominated difluoroallylbiphenyl **5c** successfully underwent the same cyclization to afford the corresponding product **6c** in 62% isolated yield. The structural characterization of **6** was achieved by X-ray analysis using a single crystal of **6a** (Figure 2), and it was found that the iodoarylation products **6** have a seven-membered carbocycle bearing adjacent difluoromethylene and iodomethylene units.

**Scheme 5:**
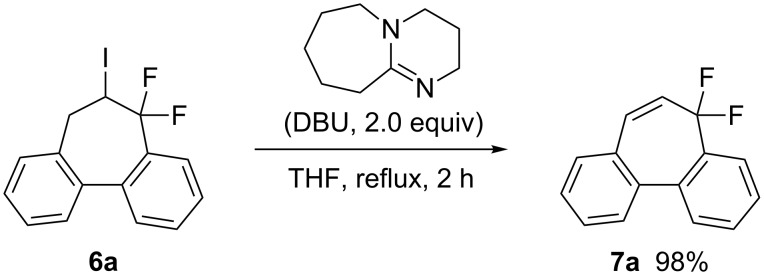
Selective HI elimination from **6a**.

In addition, a selective HI elimination from **6a** could be achieved by the choice of base, leading to the construction of a [7]annulene system (Scheme 5). The use of lithium bases, such as lithium diisopropylamide and lithium hexamethyldisilazide, induced HF eliminations as well as substantial HI elimination. However, 1,8-diazabicyclo[5.4.0]undec-7-ene (DBU) exclusively promoted HI elimination to afford ring-difluorinated dibenzo[*a*,*c*][7]annulene **7a** in an almost quantitative yield. Since dibenzo[*a*,*c*][7]annulenes serve as bioactive agents, this method would be of value in the research directed toward pharmaceutical and materials chemistry [33–35].

## Conclusion

In summary, we demonstrated selective constructions of six and seven-membered carbocyclic rings through the intramolecular iodoarylation of 3,3-difluoroallylic biaryls. The size selectivity in the cyclization was drastically controlled by the presence or absence of an aryl group in the 2-position of the 3,3-difluoroallylic moiety, which might perturb cationic charge distribution in the corresponding cyclic iodonium intermediates. The aryl group in the 2-position (at the carbon atom in β-position to the fluorine substituents) promoted a six-membered-ring closure, most likely because of the localization of cationic charge stabilized by the aromatic ring. In contrast, seven-membered carbocycles were constructed probably as a result of the cationic charge localized at the 3-position of difluoroallylic moiety (at the α-carbon atom of the fluorine substituents) due to the α-cation-stabilizing effect of fluorine.

## Experimental

**General:** ^1^H NMR, ^13^C NMR, and ^19^F NMR spectra were recorded on a Bruker Avance 500 or a JEOL ECS-400 spectrometer. Chemical shift values are given in ppm relative to internal Me_4_Si (for ^1^H NMR: δ = 0.00 ppm), CDCl_3_ (for ^13^C NMR: δ = 77.0 ppm), C_6_F_6_ (for ^19^F NMR: δ = 0.0 ppm), and (4-MeC_6_H_4_)_2_C(CF_3_)_2_ (for ^19^F NMR: δ = 97.9 ppm). IR spectra were recorded on a Horiba FT-300S spectrometer using the attenuated total reflectance (ATR) method. Mass spectra were measured on a JEOL JMS-T100GCV spectrometer. X-ray diffraction studies were performed on a Bruker APEXII ULTRA instrument equipped with a CCD diffractometer using Mo Kα (graphite monochromated, λ = 0.71069 Å) radiation. The CCDC deposition numbers of compounds **2a** and **6a** are 1556804 and 1556803, respectively. All the reactions were conducted under argon or nitrogen atmosphere.

**Materials:** Column chromatography and preparative thin-layer chromatography (PTLC) were conducted on silica gel (Silica Gel 60 N, Kanto Chemical Co., Inc. for column chromatography and Wakogel B-5F, Wako Pure Chemical Industries, Ltd. for PTLC). Tetrahydrofuran (THF), dichloromethane, and *N*,*N*-dimethylformamide (DMF) were purified by a solvent-purification system (GlassContour) equipped with columns of activated alumina and supported-copper catalyst (Q-5) before use. 1,1,1,3,3,3-Hexafluoropropan-2-ol (HFIP) was distilled from CaH_2_ and stored over activated 4 Å molecular sieves. Unless otherwise noted, materials were obtained from commercial sources and used directly without further purifications.

**Typical procedure for the iodoarylation of 2-(2-aryl-3,3-difluoroallyl)biaryls 1:** To a HFIP (1.20 mL) and dichloromethane (0.13 mL) solution of 2-(2-phenyl-3,3-difluoroallyl)biphenyl (**1a**, 31 mg, 0.10 mmol) was added pyridine iodine monochloride (PyICl, 49 mg, 0.20 mmol) at 0 °C. After stirring at the same temperature for 1 h, the reaction was quenched with an aqueous NaHCO_3_ solution. The organic materials were extracted with CHCl_3_ three times. The combined extracts were washed with an aqueous Na_2_S_2_O_3_ solution and brine, and dried over anhydrous Na_2_SO_4_. After removal of the solvent under reduced pressure, the residue was purified by PTLC (hexane/ethyl acetate 10:1) to give 9-(difluoroiodomethyl)-9-phenyl-9,10-dihydrophenanthrene (**2a**, 34 mg, 79%) as a white solid. ^1^H NMR (500 MHz, CDCl_3_) δ 3.68 (d, *J* = 15.8 Hz, 1H), 3.71 (d, *J* = 15.8 Hz, 1H), 7.07–7.08 (m, 3H), 7.15–7.24 (m, 5H), 7.42–7.49 (m, 2H), 7.52–7.54 (m, 1H), 7.79 (d, *J* = 7.5 Hz, 1H), 7.95 (d, *J* = 7.6 Hz, 1H); ^13^C NMR (126 MHz, CDCl_3_) δ 38.7, 59.5 (t, *J*_CF_ = 17 Hz), 110.6 (t, *J*_CF_ = 316 Hz), 123.6, 125.1, 127.2, 127.4, 127.50, 127.52, 128.0, 128.46, 128.50, 128.6 (t, *J*_CF_ = 4 Hz), 130.1, 132.7, 133.64, 133.64, 134.6, 136.8; ^19^F NMR (470 MHz, CDCl_3_) δ 124.7 (br s); IR (neat): 3068, 1489, 1454, 1126, 1147, 1097, 964, 850, 742, 696, 592 cm^−1^; HRMS–EI (*m*/*z*): [M]^+^ calcd for C_21_H_15_F_2_I, 432.0186; found: 432.0166.

**Typical procedure for the iodoarylation of 2-(3,3-difluoroallyl)biaryls 5:** To a HFIP (2.5 mL) and dichloromethane (1.5 mL) solution of *N*-iodosuccinimide (NIS, 27 mg, 0.12 mmol) was added trimethylsilyl trifluoromethanesulfonate (22 μL, 0.12 mmol) at 0 °C. After stirring at the same temperature for 10 min, a dichloromethane (1.0 mL) solution of 2-(3,3-difluoroallyl)biphenyl (**5a**, 23 mg, 0.10 mmol) was added to the reaction mixture. After stirring at 0 °C for 40 min, the reaction was quenched with an aqueous NaHCO_3_ solution. The organic materials were extracted with dichloromethane three times. The combined extracts were washed with an aqueous Na_2_S_2_O_3_ solution and brine, and dried over anhydrous Na_2_SO_4_. After removal of the solvent under reduced pressure, the residue was purified by PTLC (hexane/ethyl acetate 10:1) to give 5,5-difluoro-6-iodo-6,7-dihydro-5*H*-dibenzo[*a*,*c*][7]annulene (**6a**, 33 mg, 92%) as a colorless liquid. ^1^H NMR (500 MHz, CDCl_3_) δ 3.06 (dd, *J* = 14.8, 4.9, 1H), 3.38 (dd, *J* = 14.8, 6.0 Hz, 1H), 4.91–4.98 (m, 1H), 7.28–7.35 (m, 2H), 7.41–7.44 (m, 3H), 7.47 (d, *J* = 7.8 Hz, 1H), 7.55–7.59 (m, 1H), 7.70 (d, *J* = 7.4 Hz, 1H); ^13^C NMR (126 MHz, CDCl_3_) δ 35.1 (dd, *J*_CF_ = 27 Hz), 41.7, 118.9 (dd, *J*_CF_ = 247 Hz), 125.2, 127.5, 128.0, 128.20, 128.23, 129.2, 129.7, 131.0, 131.4 (dd, *J*_CF_ = 24 Hz), 134.6, 138.6 (dd, *J*_CF_ = 5 Hz), 140.3; ^19^F NMR (470 MHz, DMSO-*d*_6_, 120 °C) δ 72.3 (d, *J*_FF_ = 236 Hz, 1F), 86.5 (d, *J*_FF_ = 236 Hz, 1F); IR (neat): 3068, 3030, 1450, 1149, 1055, 989, 752, 598 cm^–1^; HRMS–EI (*m*/*z*): [M]^+^ calcd for C_15_H_11_F_2_I, 355.9873; found: 355.9866.

## Supporting Information

File 1 Detailed experimental procedures and spectral data.
