# Supplementary material for: Ring-size-selective construction of fluorine-containing carbocycles via intramolecular iodoarylation of 1,1-difluoro-1-alkenes
Source: Beilstein J Org Chem. 2017 Dec 14;13:2682–9. doi: 10.3762/bjoc.13.266 (PMC5753149; doi:10.3762/bjoc.13.266)
Supplement: File 1 — Detailed experimental procedures and spectral data. [file Beilstein_J_Org_Chem-13-2682-s001.pdf]

# Supporting Information

# for

# Ring-size-selective construction of fluorine-containing carbocycles via intramolecular iodoarylation of 1,1-difluoro-1-alkenes

Takeshi Fujita, Ryo Kinoshita, Tsuyoshi Takanohashi, Naoto Suzuki, and Junji Ichikawa*

Address: Division of Chemistry, Faculty of Pure and Applied Sciences, University of Tsukuba, Tsukuba, Ibaraki 305-8571, Japan

Email: Junji Ichikawa - junji@chem.tsukuba.ac.jp

*Corresponding author

## Detailed experimental procedures and spectral data

1. General statement ........ S2
2. Preparation of 2-(2-aryl-3,3-difluoroallyl)biaryls **1** ........ S3
3. Preparation of 2-(3,3-difluoroallyl)biaryls **5** ........ S7
4. Iodoarylation of 2-(2-aryl-3,3-difluoroallyl)biaryls **1** ........ S9
5. Synthesis of dihydrophenanthrene bearing a $CHF_2$ group ........ S13
6. Iodoarylation of 2-(3,3-difluoroallyl)biaryls **5** ........ S14
7. Synthesis of difluorodibenzo[*a*,*c*][7]annulene ........ S16
8. References ........ S17
9. $^{1}H$, $^{13}C$, and $^{19}F$ NMR charts ........ S18

## 1. General statement

$^{1}$H NMR, $^{13}$C NMR, and $^{19}$F NMR spectra were recorded on a Bruker Avance 500 or a JEOL ECS-400 spectrometer. Chemical shift values are given in ppm relative to internal $Me_4Si$ (for $^{1}$H NMR: $\delta$ = 0.00 ppm), $CDCl_3$ (for $^{13}$C NMR: $\delta$ = 77.0 ppm), $C_6F_6$ (for $^{19}$F NMR: $\delta$ = 0.0 ppm), and $(4\text{-}MeC_6H_4)_2C(CF_3)_2$ (for $^{19}$F NMR: $\delta$ = 97.9 ppm). IR spectra were recorded on a Horiba FT-300S spectrometer using the attenuated total reflectance (ATR) method. Mass spectra were measured on a JEOL JMS-T100GCV spectrometer. X-ray diffraction studies were performed on a Bruker APEXII ULTRA instrument equipped with a CCD diffractometer using MoK$\alpha$ (graphite monochromated, $\lambda$ = 0.71069 Å) radiation. The structure was solved by direct methods (SIR97) [1]. The positional and thermal parameters of non-hydrogen atoms were refined anisotropically on $F^2$ by the full-matrix least-squares method using SHELXS-97 [2]. Hydrogen atoms were placed at calculated positions and refined with the riding mode on their corresponding carbon atoms. The CCDC deposition numbers of compounds **2a** and **6a** are 1556804 and 1556803, respectively. All the reactions were conducted under argon or nitrogen atmosphere.

Column chromatography and preparative thin-layer chromatography (PTLC) were conducted on silica gel (Silica Gel 60 N, Kanto Chemical Co., Inc. for column chromatography and Wakogel B-5F, Wako Pure Chemical Inductries for PTLC). Tetrahydrofuran (THF), dichloromethane, and *N*,*N*-dimethylformamide (DMF) were purified by a solvent-purification system (GlassContour) equipped with columns of activated alumina and supported-copper catalyst (Q-5) before use. 1,1,1,3,3,3-Hexafluoropropan-2-ol (HFIP) was distilled from $CaH_2$ and stored over activated 4 Å molecular sieves. *N*,*N*,*N'*,*N'*-Tetramethylethylenediamine (TMEDA) was distilled from KOH and stored over activated 4 Å molecular sieves. 2-(Trifluoromethyl)-1-alkenes [3-5] and (biaryl-2-yl)acetaldehydes [6] were prepared according to the literature

procedures. Unless otherwise noted, materials were obtained from commercial sources and used directly without further purifications.

## 2. Preparation of 2-(2-aryl-3,3-difluoroallyl)biaryls 1

### 2-(3,3-Difluoro-2-phenylallyl)biphenyl (1a)

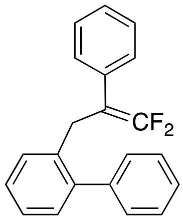


To a THF (6.0 mL) solution of 2-iodobiphenyl (105 μL, 0.60 mmol) and TMEDA (157 μL, 1.0 mmol) was added *n*-BuLi (1.58 M, 0.83 mL, 1.3 mmol) at room temperature. After stirring at the same temperature for 15 min, (3,3,3-trifluoroprop-1-en-2-yl)benzene (206 mg, 1.20 mmol) was added to the reaction mixture. After stirring at 60 °C for 2.5 h, the reaction was quenched with an aqueous $NH_4Cl$ solution. The organic materials were extracted with $CH_2Cl_2$ three times. The combined extracts were washed with brine, and dried over anhydrous $Na_2SO_4$. After removal of the solvent under reduced pressure, the residue was purified by silica gel column chromatography (hexane/ethyl acetate 50:1) to give **1a** (98 mg, 53%) as a colorless liquid.

$^1$H NMR (500 MHz, $CDCl_3$): δ 3.69 (dd, $J_{HF}$ = 2.3, 2.3 Hz, 2H), 7.06–7.07 (m, 2H), 7.14–7.28 (m, 9H), 7.32–7.39 (m, 3H). $^{13}$C NMR (126 MHz, $CDCl_3$): δ 31.2, 91.5 (dd, $J_{CF}$ = 21, 14 Hz), 126.2, 127.0, 127.1, 127.5, 128.1, 128.18, 128.21 (dd, $J_{CF}$ = 4, 4 Hz), 128.5, 129.2, 130.0, 133.2 (dd, $J_{CF}$ = 3, 3 Hz), 135.5 (dd, $J_{CF}$ = 3, 3 Hz), 141.3, 142.1, 154.2 (dd, $J_{CF}$ = 292, 289 Hz). $^{19}$F NMR (376 MHz, $CDCl_3$): δ 70.9 (d, $J_{FF}$ = 40 Hz, 1F), 71.1 (d, $J_{FF}$ = 40 Hz, 1F). IR (neat): 3060, 3022, 1724, 1477, 1236, 991, 748, 696 cm$^{-1}$. HRMS (EI): *m*/*z* Calcd for $C_{21}H_{16}F_2$ $[M]^+$: 306.1220; Found: 306.1213.

**2-[2-(Biphenyl-4-yl)-3,3-difluoroallyl]biphenyl (1b)**

Compound **1b** was prepared by the method described for **1a** using 2-iodobiphenyl (105 μL, 0.60 mmol) and 4-(3,3,3-trifluoroprop-1-en-2-yl)biphenyl (255 mg, 1.03 mmol). Purification by PTLC (hexane/ethyl acetate 10:1) gave **1b** (154 mg, 67%) as a white solid.

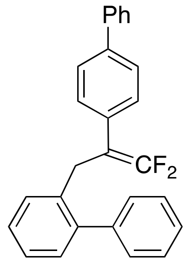


$^1$H NMR (500 MHz, $CDCl_3$): δ 3.73 (dd, $J_{HF}$ = 2.1, 2.1 Hz, 2H), 7.14 (d, $J$ = 7.5 Hz, 2H), 7.18 (dd, $J$ = 7.5, 1.6 Hz, 1H), 7.22–7.28 (m, 4H), 7.30–7.45 (m, 9H), 7.53–7.55 (m, 2H). $^{13}$C NMR (126 MHz, $CDCl_3$): δ 31.1, 91.3 (dd, $J_{CF}$ = 21, 14 Hz), 126.3, 126.87, 126.93, 127.0, 127.3, 127.6, 128.1, 128.48, 128.51 (dd, $J_{CF}$ = 4, 4 Hz), 128.7, 129.2, 130.0, 132.2 (dd, $J_{CF}$ = 4, 4 Hz), 135.5 (dd, $J_{CF}$ = 3, 2 Hz), 139.8, 140.5, 141.3, 142.1, 154.4 (dd, $J_{CF}$ = 292, 289 Hz). $^{19}$F NMR (376 MHz, $CDCl_3$): δ 71.6 (d, $J_{FF}$ = 39 Hz, 1F), 71.8 (d, $J_{FF}$ = 39 Hz, 1F). IR (neat): 3059, 3028, 1722, 1479, 1238, 999, 841, 764, 700 cm$^{-1}$. HRMS (EI): *m/z* Calcd for $C_{27}H_{20}F_2$ $[M]^+$: 382.1533; Found: 382.1549.

**2-[3,3-Difluoro-2-(4-methylphenyl)allyl]biphenyl (1c)**

Compound **1c** was prepared by the method described for **1a** using 2-iodobiphenyl (106 μL, 0.60 mmol) and 1-methyl-4-(3,3,3-trifluoroprop-1-en-2-yl)benzene (134 mg, 0.72 mmol). Purification by PTLC (hexane/ethyl acetate 10:1) gave **1c** (113 mg, 59%) as a white solid.

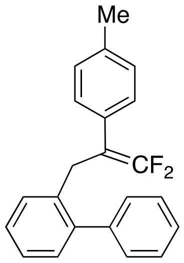


$^1$H NMR (400 MHz, $CDCl_3$): δ 2.27 (s, 3H), 3.67 (dd, $J_{HF}$ = 2.5, 2.0 Hz, 2H), 6.97 (d, $J$ = 8.3 Hz, 2H), 7.02 (d, $J$ = 8.3 Hz, 2H), 7.15–7.28 (m, 6H), 7.33–7.41 (m, 3H). $^{13}$C NMR (126 MHz, $CDCl_3$): δ 21.1, 31.1, 91.2 (dd, $J_{CF}$ = 18, 15), 126.2, 127.0, 127.5, 128.0 (dd, $J_{CF}$ = 3, 3 Hz), 128.1, 128.5, 128.9, 129.2, 129.9, 130.2, 135.6 (dd, $J_{CF}$ = 3, 2 Hz), 136.8, 141.4, 142.1, 154.2 (dd, $J_{CF}$ = 292, 290 Hz). $^{19}$F NMR (376 MHz, $CDCl_3$): δ 71.0 (d, $J_{FF}$ = 41 Hz, 1F), 71.1 (d, $J_{FF}$ = 41 Hz, 1F). IR (neat): 3022, 2922,

1722, 1479, 1236, 997, 822, 748, 702 cm$^{-1}$. HRMS (EI): *m*/*z* Calcd for $C_{22}H_{18}F_2$ [M]$^+$: 320.1377; Found: 320.1385.

**2-[3,3-Difluoro-2-(3-methylphenyl)allyl]biphenyl (1d)**

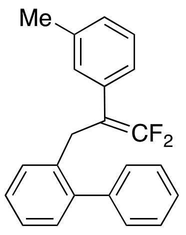


Compound **1d** was prepared by the method described for **1a** using 2-iodobiphenyl (39 μL, 0.22 mmol) and 1-methyl-3-(3,3,3-trifluoroprop-1-en-2-yl)benzene (49 mg, 0.26 mmol). Purification by PTLC (pentane) gave **1d** (14 mg, 19%) as a white solid.

$^1$H NMR (500 MHz, $CDCl_3$): δ 2.24 (s, 3H), 3.68 (dd, $J_{HF}$ = 2.4, 2.1 Hz, 2H), 6.86–6.88 (m, 2H), 6.98 (d, *J* = 7.2 Hz, 1H), 7.08–7.11 (m, 1H), 7.15–7.29 (m, 6H), 7.33–7.40 (m, 3H). $^{13}$C NMR (126 MHz, $CDCl_3$): δ 21.4, 31.1, 91.6 (dd, $J_{CF}$ = 17, 16 Hz), 125.3 (dd, $J_{CF}$ = 4, 3 Hz), 126.2, 126.9, 127.5, 127.9, 128.0, 128.1, 128.5, 128.9 (dd, $J_{CF}$ = 4, 3), 129.2, 129.9, 133.1, 135.6 (dd, $J_{CF}$ = 3, 3 Hz), 137.7, 141.4, 142.1, 154.2 (dd, $J_{CF}$ = 290, 290 Hz). $^{19}$F NMR (470 MHz, $CDCl_3$): δ 70.9 (d, $J_{FF}$ = 41 Hz, 1F), 71.0 (d, $J_{FF}$ = 41 Hz, 1F). IR (neat): 3060, 2924, 1730, 1479, 1244, 1120, 1018, 787, 748, 702 cm$^{-1}$. HRMS (EI): *m*/*z* Calcd for $C_{22}H_{18}F_2$ [M]$^+$: 320.1377; Found: 320.1376.

**2-[3,3-Difluoro-2-(4-methoxyphenyl)allyl]biphenyl (1e)**

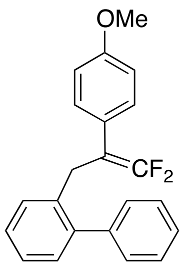


Compound **1e** was prepared by the method described for **1a** using 2-iodobiphenyl (88 μL, 0.50 mmol) and 1-methoxy-4-(3,3,3-trifluoroprop-1-en-2-yl)benzene (121 mg, 0.60 mmol). Purification by PTLC (hexane/ethyl acetate 10:1) gave **1e** (92 mg, 55%) as a pale yellow liquid.

$^1$H NMR (500 MHz, $CDCl_3$): δ 3.66 (dd, $J_{HF}$ = 2.1, 2.0 Hz, 2H), 3.71 (s, 3H), 6.73 (d, *J* = 8.8 Hz, 2H), 6.98 (d, *J* = 8.8 Hz, 2H), 7.14–7.27 (m, 6H), 7.31–7.39 (m, 3H). $^{13}$C

NMR (126 MHz, $CDCl_3$): δ 31.2, 55.1, 91.0 (dd, $J_{CF}$ = 19, 16 Hz), 113.6, 125.4, 126.2, 126.9, 127.5, 128.1, 128.5, 129.2, 129.3 (dd, $J_{CF}$ = 4, 3 Hz), 129.9, 135.6 (dd, $J_{CF}$ = 2, 2 Hz), 141.4, 142.1, 154.1 (dd, $J_{CF}$ = 289, 289 Hz), 158.5. $^{19}F$ NMR (376 MHz, $CDCl_3$): δ 70.0 (br s, 2F). IR (neat): 3020, 2837, 1726, 1610, 1514, 1296, 1238, 1180, 995, 833, 748, 704 $cm^{-1}$. HRMS (EI): *m*/*z* Calcd for $C_{22}H_{18}F_2O$ $[M]^+$: 336.1326; Found: 336.1311.

**2-(3,3-Difluoro-2-phenylallyl)-4'-methylbiphenyl (1f)**

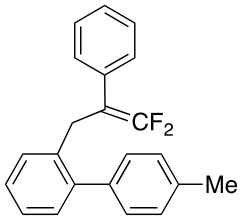


Compound **1f** was prepared by the method described for **1a** using 2-bromo-4'-methylbiphenyl (148 mg, 0.60 mmol) and (3,3,3-trifluoroprop-1-en-2-yl)benzene (123 mg, 0.72 mmol). Purification by PTLC (hexane/ethyl acetate 10:1) gave **1f** (74 mg, 38%) as a colorless liquid.

$^1H$ NMR (500 MHz, $CDCl_3$): δ 2.40 (s, 3H), 3.69 (dd, $J_{HF}$ = 2.1, 2.1 Hz, 2H), 7.07–7.10 (m, 4H), 7.14–7.27 (m, 9H). $^{13}C$ NMR (100 MHz, $CDCl_3$): δ 21.2, 31.1, 91.5 (dd, $J_{CF}$ = 21, 14 Hz), 126.2, 127.1, 127.4, 128.18, 128.21 (dd, $J_{CF}$ = 3, 3 Hz), 128.4, 128.8, 129.1, 130.1, 133.3 (dd, $J_{CF}$ = 3, 3 Hz), 135.6, 136.6, 138.4, 142.1, 154.3 (dd, $J_{CF}$ = 290, 287 Hz). $^{19}F$ NMR (470 MHz, $CDCl_3$): δ 71.4 (d, $J_{FF}$ = 40 Hz, 1F), 71.6 (d, $J_{FF}$ = 40 Hz, 1F). IR (neat): 3024, 2922, 1726, 1481, 1446, 1238, 1005, 991, 758, 696 $cm^{-1}$. HRMS (EI): *m*/*z* Calcd for $C_{22}H_{18}F_2$ $[M]^+$: 320.1377; Found: 320.1362.

**2-(3,3-Difluoro-2-phenylallyl)-4'-methoxybiphenyl (1g)**

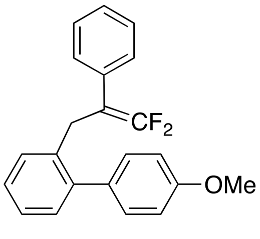


Compound **1g** was prepared by the method described for **1a** using 2-bromo-4'-methoxybiphenyl (158 mg, 0.60 mmol) and (3,3,3-trifluoroprop-1-en-2-yl)benzene (123 mg, 0.72 mmol).

Purification by PTLC (hexane/ethyl acetate 30:1) gave **1g** (15 mg, 7%) as a colorless liquid.

$^1$H NMR (400 MHz, $CDCl_3$): δ 3.69 (br s, 2H), 3.85 (s, 3H), 6.92 (d, $J$ = 8.0 Hz, 2H), 7.06–7.27 (m, 11H). $^{13}$C NMR (100 MHz, $CDCl_3$): δ 30.8, 55.2, 91.6 (dd, $J_{CF}$ = 20, 14 Hz), 113.6, 126.2, 127.1, 127.3, 128.18, 128.21 (dd, $J_{CF}$ = 4, 4 Hz), 128.5, 130.2, 130.3, 133.3 (dd, $J_{CF}$ = 3, 3 Hz), 133.7, 135.7, 141.8, 154.3 (dd, $J_{CF}$ = 290, 287 Hz), 158.7. $^{19}$F NMR (376 MHz, $CDCl_3$): δ 72.0 (d, $J_{FF}$ = 40 Hz, 1F), 72.1 (d, $J_{FF}$ = 40 Hz, 1F). IR (neat): 3064, 2958, 2837, 1726, 1612, 1516, 1481, 1242, 1003, 835, 762 $cm^{-1}$. HRMS (EI): $m/z$ Calcd for $C_{22}H_{18}F_2O$ $[M]^+$: 336.1326; Found: 336.1317.

### 3. Preparation of 2-(3,3-difluoroallyl)biaryls 5

#### 2-(3,3-Difluoroallyl)biphenyl (5a)

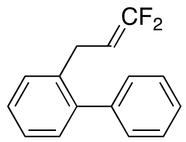


To a THF (130 mL) suspension of molecular sieves (4 Å, powder, 4.2 g) was added dibromodifluoromethane (5.9 mL, 64 mmol) at −78 °C. After stirring at the same temperature for 30 min, tris(dimethylamino)phosphine (23.4 mL, 129 mmol) was added dropwise to the mixture at −78 °C. After stirring at the same temperature for another 35 min, the reaction mixture was allowed to warm slowly to room temperature, and a THF (20 mL) solution of 2-(biphenyl-2-yl)acetaldehyde (4.23 g, 21.6 mmol) was added slowly to the reaction mixture. After stirring at room temperature for 10 h, the reaction mixture was filtered through a pad of silica gel (ethyl acetate). After removal of the solvent under reduced pressure, the residue was purified by silica gel column chromatography (hexane) to give **5a** (2.56 g, 52%) as a colorless liquid.

$^1$H NMR (500 MHz, $CDCl_3$): δ 3.27 (ddd, $J$ = 7.9 Hz, $J_{HF}$ = 1.7, 1.7 Hz, 2H), 4.21 (dtd, $J_{HF}$ = 24.9 Hz, $J$ = 7.9 Hz, $J_{HF}$ = 2.4 Hz, 1H), 7.22–7.24 (m, 1H), 7.26–7.38 (m, 6H),

7.40–7.44 (m, 2H). $^{13}C$ NMR (126 MHz, $CDCl_3$): δ 26.1 (d, $J_{CF}$ = 5 Hz), 77.8 (dd, $J_{CF}$ = 23, 20 Hz), 126.4, 127.0, 127.6, 128.1, 128.8, 129.0, 130.0, 136.8, 141.2, 141.8, 156.2 (dd, $J_{CF}$ = 288, 286 Hz). $^{19}F$ NMR (376 MHz, $CDCl_3$): δ 70.2 (dd, $J_{FF}$ = 45 Hz, $J_{FH}$ = 25 Hz, 1F), 72.7 (d, $J_{FF}$ = 45 Hz, 1F). IR (neat): 3060, 2925, 1745, 1479, 1225, 1174, 752, 702 $cm^{-1}$. HRMS (EI): *m*/*z* Calcd for $C_{15}H_{12}F_2$ $[M]^+$: 230.0907; Found: 230.0902.

**2-(3,3-Difluoroallyl)-4'-methylbiphenyl (5b)**

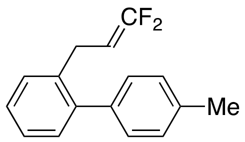


Compound **5b** was prepared by the method described for **5a** using 2-(4'-methylbiphenyl-2-yl)acetaldehyde (526 mg, 2.50 mmol). Purification by silica gel column chromatography (hexane) gave **5b** (248 mg, 41%) as a colorless liquid.

$^1H$ NMR (500 MHz, $CDCl_3$): δ 2.40 (s, 3H), 3.27 (ddd, *J* = 7.9 Hz, $J_{HF}$ = 1.8, 1.8 Hz, 2H), 4.22 (dtd, $J_{HF}$ = 25.0 Hz, *J* = 7.9 Hz, $J_{HF}$ = 2.3 Hz, 1H), 7.16–7.19 (m, 2H), 7.20–7.32 (m, 6H). $^{13}C$ NMR (126 MHz, $CDCl_3$): δ 21.1, 26.1 (d, $J_{CF}$ = 5 Hz), 77.8 (dd, $J_{CF}$ = 23, 20 Hz), 126.4, 127.4, 128.8, 128.85, 128.91, 130.1, 136.7, 136.9 (dd, $J_{CF}$ = 2, 2 Hz), 138.3, 141.7, 156.3 (dd, $J_{CF}$ = 288, 286 Hz). $^{19}F$ NMR (376 MHz, $CDCl_3$): δ 70.1 (dd, $J_{FF}$ = 45 Hz, $J_{FH}$ = 25 Hz, 1F), 72.7 (d, $J_{FF}$ = 45 Hz, 1F). IR (neat): 3024, 2924, 1745, 1483, 1225, 1173, 758 $cm^{-1}$. HRMS (EI): *m*/*z* Calcd for $C_{16}H_{14}F_2$ $[M]^+$: 244.1064; Found: 244.1052.

**4'-Bromo-2-(3,3-difluoroallyl)biphenyl (5c)**

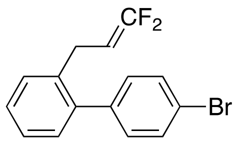


Compound **5c** was prepared by the method described for **5a** using 2-(4'-bromobiphenyl-2-yl)acetaldehyde (1.18 g, 4.29 mmol). Purification by silica gel column chromatography (hexane) gave

**5c** (438 mg, 33%) as a pale yellow liquid.

$^1$H NMR (400 MHz, $CDCl_3$): δ 3.24 (d, $J$ = 7.6 Hz, 2H), 4.15–4.26 (m, 1H), 7.12–7.20 (m, 3H), 7.25–7.42 (m, 3H), 7.54–7.56 (m, 2H). $^{13}$C NMR (100 MHz, $CDCl_3$): δ 26.1 (d, $J_{CF}$ = 4 Hz), 77.7 (dd, $J_{CF}$ = 23, 22 Hz), 121.3, 126.6, 128.0, 129.0, 130.0, 130.8, 131.4, 136.8, 140.2, 140.5, 156.3 (dd, $J_{CF}$ = 286, 286 Hz). $^{19}$F NMR (376 MHz, $CDCl_3$): δ 70.9 (dd, $J_{FF}$ = 45 Hz, $J_{FH}$ = 25 Hz, 1F), 73.4 (d, $J_{FF}$ = 45 Hz, 1F). IR (neat): 3064, 2931, 1743, 1475, 1225, 1072, 1005, 829, 756 cm$^{-1}$. HRMS (EI): $m/z$ Calcd for $C_{15}H_{11}{}^{79}BrF_2$ $[M]^+$: 308.0012; Found: 307.9998.

**4. Iodoarylation of 2-(2-aryl-3,3-difluoroallyl)biaryls 1**

**9-(Difluoroiodomethyl)-9-phenyl-9,10-dihydrophenanthrene (2a)**

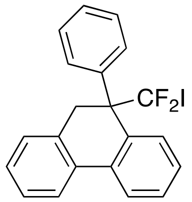


To a HFIP (1.20 mL) and dichloromethane (0.13 μL) solution of 2-(2-phenyl-3,3-difluoroallyl)biphenyl (**1a**, 31 mg, 0.10 mmol) was added pyridine iodine monochloride (PyICl, 49 mg, 0.20 mmol) at 0 °C. After stirring at 0 °C for 1 h, the reaction was quenched with an aqueous $NaHCO_3$ solution. The organic materials were extracted with $CHCl_3$ three times. The combined extracts were washed with an aqueous $Na_2S_2O_3$ solution and brine, and dried over anhydrous $Na_2SO_4$. After removal of the solvent under reduced pressure, the residue was purified by PTLC (hexane/ethyl acetate 10:1) to give **2a** (34 mg, 79%) as a white solid.

$^1$H NMR (500 MHz, $CDCl_3$): δ 3.68 (d, $J$ = 15.8 Hz, 1H), 3.71 (d, $J$ = 15.8 Hz, 1H), 7.07–7.08 (m, 3H), 7.15–7.24 (m, 5H), 7.42–7.49 (m, 2H), 7.52–7.54 (m, 1H), 7.79 (d, $J$ = 7.5 Hz, 1H), 7.95 (d, $J$ = 7.6 Hz, 1H). $^{13}$C NMR (126 MHz, $CDCl_3$): δ 38.7, 59.5 (t, $J_{CF}$ = 17 Hz), 110.6 (t, $J_{CF}$ = 316 Hz), 123.6, 125.1, 127.2, 127.4, 127.50, 127.52, 128.0, 128.46, 128.50, 128.6 (t, $J_{CF}$ = 4 Hz), 130.1, 132.7, 133.64, 133.64, 134.6,

136.8. $^{19}$F NMR (470 MHz, $CDCl_3$): δ 124.7 (br s). IR (neat): 3068, 1489, 1454, 1126, 1147, 1097, 964, 850, 742, 696, 592 cm$^{-1}$. HRMS (EI): *m/z* Calcd for $C_{21}H_{15}F_2I$ $[M]^+$: 432.0186: Found: 432.0166.

**9-(Biphenyl-4-yl)-9-(difluoroiodomethyl)-9,10-dihydrophenanthrene (2b)**

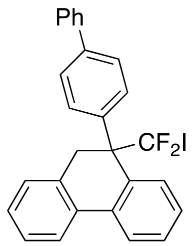


Compound **2b** was synthesized by the method described for **2a** using 2-(2-aryl-3,3-difluoroallyl)biaryl **1b** (38 mg, 0.10 mmol). Purification by PTLC (hexane/ethyl acetate 6:1) gave **2b** (38 mg, 74%) as a white solid.

$^1$H NMR (500 MHz, $CDCl_3$): δ 3.73 (br s, 2H), 7.17–7.18 (m, 2H), 7.22–7.35 (m, 8H), 7.42–7.51 (m, 4H), 7.55–7.57 (m, 1H), 7.82 (d, *J* = 7.3 Hz, 1H), 7.97 (d, *J* = 7.9 Hz, 1H). $^{13}$C NMR (126 MHz, $CDCl_3$): δ 38.8, 59.4 (t, $J_{CF}$ = 18 Hz), 110.6 (t, $J_{CF}$ = 316 Hz), 123.6, 125.1, 126.1, 126.9, 127.27, 127.32, 127.4, 128.1, 128.5, 128.65, 128.65, 128.65, 130.5, 132.66, 132.66, 133.6, 134.6, 136.8, 140.1, 140.2. $^{19}$F NMR (470 MHz, $CDCl_3$): δ 124.7 (br s, 2F). IR (neat): 3064, 3032, 1487, 1452, 1147, 1095, 1007, 964, 876, 762, 704 cm$^{-1}$. HRMS (EI): *m/z* Calcd for $C_{27}H_{19}F_2I$ $[M]^+$: 508.0500: Found: 508.0480.

**9-(Difluoroiodomethyl)-9-(4-methylphenyl)-9,10-dihydrophenanthrene (2c)**

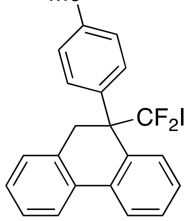


Compound **2c** was synthesized by the method described for **2a** using 2-(2-aryl-3,3-difluoroallyl)biaryl **1c** (117 mg, 0.37 mmol). Purification by silica gel column chromatography (hexane/ethyl acetate 10:1) gave **2c** (134 mg, 82%) as a white solid.

$^1$H NMR (400 MHz, $CDCl_3$): δ 2.12 (s, 3H), 3.66 (s, 2H), 6.85 (d, *J* = 8.2 Hz, 2H), 7.04 (d, *J* = 8.2 Hz, 2H), 7.11–7.16 (m, 2H), 7.20–7.23 (m, 1H), 7.38–7.46 (m, 2H), 7.50–

7.53 (m, 1H), 7.77 (dd, $J$ = 7.6, 1.6 Hz, 1H), 7.93 (d, $J$ = 7.2 Hz, 1H). $^{13}$C NMR (100 MHz, $CDCl_3$): δ 20.9, 38.7, 59.3 (t, $J_{CF}$ = 17 Hz), 111.0 (t, $J_{CF}$ = 317 Hz), 123.5, 125.0, 127.2, 127.3, 128.0, 128.3, 128.4, 128.5, 128.6 (t, $J_{CF}$ = 4 Hz), 130.0, 132.81, 132.81, 133.6, 134.5, 136.9, 137.2. $^{19}$F NMR (376 MHz, $CDCl_3$): δ 125.2 (br s, 2F). IR (neat): 3026, 2922, 1512, 1454, 1149, 1126, 1095, 964, 874, 856, 746 $cm^{-1}$. HRMS (EI): *m*/*z* Calcd for $C_{22}H_{17}F_2I$ $[M]^+$: 446.0343; Found: 446.0322.

**9-(Difluoroiodomethyl)-9-(3-methylphenyl)-9,10-dihydrophenanthrene (2d)**

Compound **2d** was synthesized by the method described for **2a** using 2-(2-aryl-3,3-difluoroallyl)biaryl **1d** (13 mg, 42 μmol). Purification by PTLC (hexane/ethyl acetate 10:1) gave **2d** (10 mg, 53%) as a white solid.

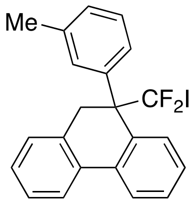


$^1$H NMR (500 MHz, $CDCl_3$): δ 2.16 (s, 3H), 3.66 (d, $J$ = 15.4 Hz, 1H), 3.70 (d, $J$ = 15.4 Hz, 1H), 6.90–6.97 (m, 4H), 7.15–7.19 (m, 2H), 7.24–7.25 (m, 1H), 7.43 (ddd, $J$ = 7.5, 7.5, 1.6 Hz, 1H), 7.48 (ddd, $J$ = 7.7, 7.7, 1.5 Hz, 1H), 7.53–7.56 (m, 1H), 7.80 (dd, $J$ = 7.7, 1.5 Hz, 1H), 7.93 (d, $J$ = 8.8 Hz, 1H). $^{13}$C NMR (100 MHz, $CDCl_3$): δ 21.5, 38.8, 59.4 (t, $J_{CF}$ = 17 Hz), 110.9 (t, $J_{CF}$ = 318 Hz), 123.5, 125.0, 127.1, 127.28, 127.31, 127.31, 127.9, 128.32, 128.39, 128.4, 128.7, 130.9, 132.8, 133.4, 133.6, 134.6, 136.87, 136.87. $^{19}$F NMR (376 MHz, $CDCl_3$): δ 126.0 (br s, 2F). IR (neat): 3064, 2970, 2362, 1489, 1452, 1126, 964, 845, 737 $cm^{-1}$. HRMS (EI): *m*/*z* Calcd for $C_{22}H_{17}F_2I$ $[M]^+$: 446.0343; Found: 446.0360.

**9-(Difluoroiodomethyl)-9-(4-methoxyphenyl)-9,10-dihydrophenanthrene (2e)**

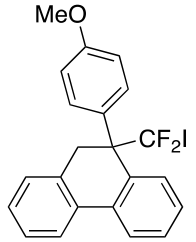


Compound **2e** was synthesized by the method described for **2a** using 2-(2-aryl-3,3-difluoroallyl)biaryl **1e** (34 mg, 0.10 mmol). Purification by PTLC (hexane/ethyl acetate 5:1) gave **2e** (38 mg, 83%) as a white solid.

$^1$H NMR (500 MHz, $CDCl_3$): δ 3.64 (s, 3H), 3.66 (br s, 2H), 6.60 (d, *J* = 8.9 Hz, 2H), 7.07 (d, *J* = 8.9 Hz, 2H), 7.16–7.19 (m, 2H), 7.23–7.26 (m, 1H), 7.41–7.48 (m, 2H), 7.53–7.55 (m, 1H), 7.80 (d, *J* = 7.7 Hz, 1H), 7.93 (d, *J* = 7.7 Hz, 1H). $^{13}$C NMR (100 MHz, $CDCl_3$): δ 38.7, 55.0, 59.0 (t, $J_{CF}$ = 17 Hz), 111.0 (t, $J_{CF}$ = 322 Hz), 112.9, 123.5, 125.1, 127.2, 127.3, 128.0, 128.4, 128.49, 128.49, 131.3, 132.81, 132.81, 133.6, 134.5, 136.9, 158.7. $^{19}$F NMR (470 MHz, $CDCl_3$): δ 124.5 (br s, 2F). IR (neat): 3064, 2837, 1606, 1510, 1454, 1259, 1188, 1093, 964, 804, 752 $cm^{-1}$. HRMS (EI): *m*/*z* Calcd for $C_{22}H_{17}F_2IO$ $[M]^+$: 462.0292; Found: 462.0293.

**10-(Difluoroiodomethyl)-2-methyl-10-phenyl-9,10-dihydrophenanthrene (2f)**

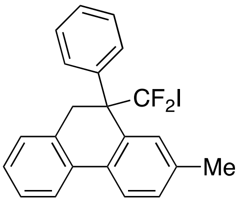


Compound **2f** was synthesized by the method described for **2a** using 2-(2-aryl-3,3-difluoroallyl)biaryl **1f** (67 mg, 0.21 mmol). Purification by PTLC (hexane/ethyl acetate 10:1) gave **2f** (50 mg, 54%) as a colorless liquid.

$^1$H NMR (500 MHz, $CDCl_3$): δ 2.49 (s, 3H), 3.64 (d, *J* = 15.1 Hz, 1H), 3.69 (d, *J* = 15.1 Hz, 1H), 7.04–7.08 (m, 3H), 7.11–7.13 (m, 2H), 7.17–7.21 (m, 3H), 7.27 (d, *J* = 7.9 Hz, 1H), 7.47–7.50 (m, 1H), 7.67 (d, *J* = 7.9 Hz, 1H), 7.74 (s, 1H). $^{13}$C NMR (126 MHz, $CDCl_3$): δ 21.7, 38.9, 59.5 (t, $J_{CF}$ = 17 Hz), 110.8 (t, $J_{CF}$ = 315 Hz), 123.2, 124.9, 127.3, 127.47, 127.47, 127.54, 128.4, 129.1, 129.3, 130.1, 131.9, 132.4, 133.7, 136.7, 136.94, 136.94. $^{19}$F NMR (376 MHz, $CDCl_3$): δ 126.1 (br s, 2F). IR

(neat): 3033, 2918, 1487, 1448, 1151, 1128, 1099, 974, 841, 766, 739, 694 $cm^{-1}$. HRMS (EI): *m*/*z* Calcd for $C_{22}H_{17}F_2I$ $[M]^+$: 446.0343; Found: 446.0322.

**10-(Difluoroiodomethyl)-2-methoxy-10-phenyl-9,10-dihydrophenanthrene (2g)**

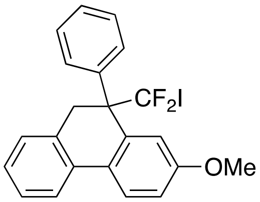


Compound **2g** was synthesized by the method described for **2a** using 2-(2-aryl-3,3-difluoroallyl)biaryl **1g** (12 mg, 37 μmol). Purification by PTLC (hexane/ethyl acetate 10:1) gave **2g** (14 mg, 80%) as a white solid.

$^1$H NMR (500 MHz, $CDCl_3$): δ 3.64 (d, $J$ = 14.9 Hz, 1H), 3.69 (d, $J$ = 14.9 Hz, 1H), 3.94 (s, 3H), 7.02 (dd, $J$ = 8.7, 2.5 Hz, 1H), 7.07–7.15 (m, 5H), 7.19–7.23 (m, 3H), 7.45–7.47 (m, 1H), 7.51 (d, $J$ = 2.5 Hz, 1H), 7.73 (d, $J$ = 8.7 Hz, 1H). $^{13}$C NMR (100 MHz, $CDCl_3$): δ 38.8, 55.5, 59.6 (t, $J_{CF}$ = 17 Hz), 110.4 (t, $J_{CF}$ = 321 Hz), 113.5, 115.0 (t, $J_{CF}$ = 3.8 Hz) 122.9, 126.2, 127.1, 127.3, 127.5, 127.57, 127.57, 127.57, 128.4, 130.1, 131.9, 133.6, 138.1, 158.6. $^{19}$F NMR (470 MHz, $CDCl_3$): δ 125.0 (br s, 2F). IR (neat): 3060, 2960, 1566, 1487, 1454, 1230, 1097, 1043, 972, 841, 729 $cm^{-1}$. HRMS (EI): *m*/*z* Calcd for $C_{22}H_{17}F_2IO$ $[M]^+$: 462.0292; Found: 462.0291.

**5. Synthesis of dihydrophenanthrene bearing a $CHF_2$ group**

**9-(Difluoromethyl)-9-phenyl-9,10-dihydrophenanthrene (4a)**

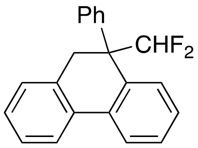


After refluxing a DMF (1.0 mL) solution of **2a** (41 mg, 96 μmol) for 15 h, the organic materials were extracted with a hexane/ethyl acetate 1:1 mixed solvent three times. The combined extracts were washed with brine, and dried over anhydrous $Na_2SO_4$. After removal of the solvent under reduced pressure, the residue was purified by silica gel column

chromatography (hexane/ethyl acetate 10:1) to give **4a** (29 mg, 97%) as a colorless liquid.

$^1$H NMR (400 MHz, $CDCl_3$): δ 3.36 (d, *J* = 15.6 Hz, 1H), 3.58 (d, *J* = 15.6 Hz, 1H), 6.25 (t, $J_{HF}$ = 55.8 Hz, 1H), 7.08–7.24 (m, 8H), 7.34–7.49 (m, 3H), 7.63 (d, *J* = 7.2 Hz, 1H), 7.83 (d, *J* = 7.6 Hz, 1H). $^{13}$C NMR (100 MHz, $CDCl_3$): δ 35.0 (t, $J_{CF}$ = 5 Hz), 50.8 (t, $J_{CF}$ = 19 Hz), 118.5 (t, $J_{CF}$ = 248 Hz), 123.6, 124.9, 127.0, 127.3, 127.6, 127.8, 127.9, 128.0, 128.2, 128.7, 128.8, 133.1, 133.6, 134.8, 136.5, 138.2. $^{19}$F NMR (470 MHz, $CDCl_3$): δ 36.5 (dd, $J_{FF}$ = 274 Hz, $J_{FH}$ = 56 Hz, 1F), 41.3 (dd, $J_{FF}$ = 274 Hz, $J_{FH}$ = 56 Hz, 1F). IR (neat): 3064, 2970, 1489, 1454, 1124, 1065, 741, 698 cm$^{-1}$. HRMS (EI): *m*/*z* Calcd for $C_{21}H_{16}F_2$ $[M]^+$: 306.1220; Found: 306.1211.

**6. Iodoarylation of 2-(3,3-difluoroallyl)biaryls 5**

**5,5-Difluoro-6-iodo-6,7-dihydro-5*H*-dibenzo[*a*,*c*][7]annulene (6a)**

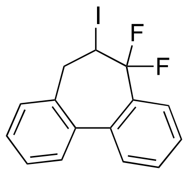


To a HFIP (2.5 mL) and dichloromethane (1.5 μL) solution of *N*-iodosuccinimide (NIS, 27 mg, 0.12 mmol) was added trimethylsilyl trifluoromethanesulfonate (22 μL, 0.12 mmol) at 0 °C. After stirring at the same temperature for 10 min, a dichloromethane (1.0 mL) solution of 2-(3,3-difluoroallyl)biphenyl (**5a**, 23 mg, 0.10 mmol) was added to the reaction mixture. After stirring at 0 °C for 40 min, the reaction was quenched with an aqueous $NaHCO_3$ solution. The organic materials were extracted with dichloromethane three times. The combined extracts were washed with an aqueous $Na_2S_2O_3$ solution and brine, and dried over anhydrous $Na_2SO_4$. After removal of the solvent under reduced pressure, the residue was purified by PTLC (hexane/ethyl acetate 10:1) to give **6a** (33 mg, 92%) as a colorless liquid.

$^{1}$H NMR (500 MHz, $CDCl_3$): δ 3.06 (dd, $J$ = 14.8, 4.9, 1H), 3.38 (dd, $J$ = 14.8, 6.0 Hz, 1H), 4.91–4.98 (m, 1H), 7.28–7.35 (m, 2H), 7.41–7.44 (m, 3H), 7.47 (d, $J$ = 7.8 Hz, 1H), 7.55–7.59 (m, 1H), 7.70 (d, $J$ = 7.4 Hz, 1H). $^{13}$C NMR (126 MHz, $CDCl_3$): δ 35.1 (dd, $J_{CF}$ = 27, 27 Hz), 41.7, 118.9 (dd, $J_{CF}$ = 247, 247 Hz), 125.2, 127.5, 128.0, 128.20, 128.23, 129.2, 129.7, 131.0, 131.4 (dd, $J_{CF}$ = 24, 24 Hz), 134.6, 138.6 (dd, $J_{CF}$ = 5, 5 Hz), 140.3. $^{19}$F NMR (376 MHz, DMSO-$d_6$, 120 °C): δ 72.3 (d, $J_{FF}$ = 236 Hz, 1F), 86.5 (d, $J_{FF}$ = 236 Hz, 1F). IR (neat): 3068, 3030, 1450, 1149, 1055, 989, 752, 598 cm$^{-1}$. HRMS (EI): $m/z$ Calcd for $C_{15}H_{11}F_2I$ [M]$^+$: 355.9873; Found: 355.9866.

**5,5-Difluoro-6-iodo-3-methyl-6,7-dihydro-5*H*-dibenzo[*a*,*c*][7]annulene (6b)**

Compound **6b** was synthesized by the method described for **6a** using 2-(3,3-difluoroallyl)biaryl **5b** (98 mg, 0.40 mmol). Purification by PTLC (hexane/ethyl acetate 10:1) gave **6b** (89 mg, 60%) as a colorless oil.

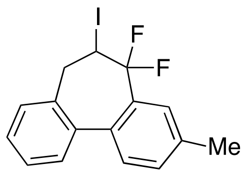


$^{1}$H NMR (400 MHz, $CDCl_3$): δ 2.46 (s, 3H), 3.06 (dd, $J$ = 14.7, 5.4 Hz, 1H), 3.38 (dd, $J$ = 14.7, 6.0 Hz, 1H), 4.89–4.98 (m, 1H), 7.27–7.35 (m, 2H), 7.37–7.42 (m, 4H), 7.51 (br s, 1H). $^{13}$C NMR (100 MHz, $CDCl_3$): δ 21.3, 35.2 (dd, $J_{CF}$ = 28, 28 Hz), 41.8, 119.0 (dd, $J_{CF}$ = 245, 245 Hz), 125.9 (dd, $J_{CF}$ = 7, 7 Hz), 127.8, 128.1, 128.2, 129.2, 129.6, 131.3, 131.6, 134.6, 135.7 (dd, $J_{CF}$ = 5, 5 Hz), 137.6, 140.4. $^{19}$F NMR (376 MHz, DMSO-$d_6$, 120 °C): δ 72.3 (d, $J_{FF}$ = 234 Hz, 1F), 86.6 (d, $J_{FF}$ = 234 Hz, 1F). IR (neat): 3030, 2952, 1481, 1448, 1184, 1151, 1043, 829, 758 cm$^{-1}$. HRMS (EI): $m/z$ Calcd for $C_{16}H_{13}F_2I$ [M]$^+$: 370.0030; Found: 370.0040.

**3-Bromo-5,5-difluoro-6-iodo-6,7-dihydro-5*H*-dibenzo[*a*,*c*][7]annulene (6c)**

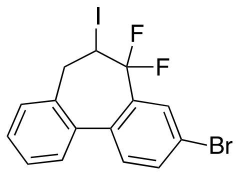


Compound **6c** was synthesized by the method described for **6a** using 2-(3,3-difluoroallyl)biaryl **5c** (62 mg, 0.20 mmol). Purification by PTLC (hexane/ethyl acetate 10:1) gave **6c** (54 mg, 62%) as a colorless oil.

$^{1}$H NMR (400 MHz, $CDCl_3$): δ 3.07 (dd, *J* = 14.8, 5.2, 1H), 3.38 (dd, *J* = 14.8, 6.4 Hz, 1H), 4.88–4.98 (m, 1H), 7.29 (d, *J* = 6.8 Hz, 1H), 7.33–7.46 (m, 4H), 7.71 (dd, *J* = 8.2, $J_{HF}$ = 2.0 Hz, 1H), 7.85 (d, *J* = 2.0 Hz, 1H). $^{13}$C NMR (100 MHz, $CDCl_3$): δ 34.2 (dd, $J_{CF}$ = 28, 28 Hz), 41.6, 118.2 (dd, $J_{CF}$ = 247, 247 Hz), 121.6, 128.1, 128.39, 128.43, 128.5 (dd, $J_{CF}$ = 8, 8 Hz), 129.4, 131.2, 133.2 (dd, $J_{CF}$ = 25, 25 Hz), 134.1, 134.5, 137.6 (dd, $J_{CF}$ = 4, 4 Hz), 139.2. $^{19}$F NMR (376 MHz, DMSO-$d_6$, 120 °C): δ 71.7 (d, $J_{FF}$ = 240 Hz, 1F), 85.9 (d, $J_{FF}$ = 240 Hz, 1F). IR (neat): 3064, 2960, 1473, 1448, 1209, 1153, 1057, 1043, 1003, 831, 756 cm$^{-1}$. HRMS (EI): *m*/*z* Calcd for $C_{15}H_{10}{}^{81}BrF_2I$ $[M]^+$: 435.8958; Found: 435.8943.

## 7. Synthesis of difluorodibenzo[*a*,*c*][7]annulene

**5,5-Difluoro-5*H*-dibenzo[*a*,*c*][7]annulene (7a)**

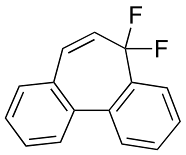


To a THF (2.6 mL) solution of **6a** (94 mg, 0.26 mmol) was added diazabicyclo[5.4.0]undec-7-ene (80 μL, 0.52 mmol). After being refluxed for 2 h, the reaction mixture was cooled to room temperature and an aqueous $NH_4Cl$ solution was added. The organic materials were extracted with $CHCl_3$ three times. The combined extracts were washed with brine, and dried over anhydrous $Na_2SO_4$. After removal of the solvent under reduced pressure, **7a** (59 mg, 98%) was obtained as a colorless liquid.

$^{1}$H NMR (500 MHz, $CDCl_3$): δ 6.28 (dt, $J$ = 10.8 Hz, $J_{HF}$ = 10.5 Hz, 1H), 6.81 (d, $J$ = 10.8 Hz, 1H), 7.32–7.47 (m, 5H), 7.63–7.74 (m, 3H). $^{13}$C NMR (126 MHz, $CDCl_3$): δ 116.4 (t, $J_{CF}$ = 240 Hz), 122.1 (t, $J_{CF}$ = 8 Hz), 127.5, 127.8, 128.0, 129.38, 129.46 (t, $J_{CF}$ = 34 Hz), 129.49, 129.8, 131.1, 132.9 (t, $J_{CF}$ = 10 Hz), 133.1, 135.4 (t, $J_{CF}$ = 4 Hz), 137.0 (t, $J_{CF}$ = 28 Hz), 138.3. $^{19}$F NMR (376 MHz, DMSO-$d_6$, 120 °C): δ 65.7 (br s, 2F). IR (neat): 3064, 2931, 1645, 1487, 1446, 1294, 1159, 1043, 1001, 764, 737 $cm^{-1}$.

## 8. References


1. Altomare, A.; Burla, M. C.; Camalli, M.; Cascarano, G. L.; Giacovazzo, C.; Guagliardi, A.; Moliterni, A. G. G.; Polidori, G.; Spagna, R. *J. Appl. Crystallogr.,* **1999,** *32,* 115–119.

2. Sheldrick, G. M. *Programs for Crystal Structure Analysis (Release 972);* Institut für Anorganische Chemie der Universität: Göttingen, Germany, 1998.

3. Ichitsuka, T.; Fujita, T.; Arita, T.; Ichikawa, J. *Angew. Chem. Int. Ed.,* **2014,** *53,* 7564–7568.

4. Ichitsuka, T.; Fujita, T.; Ichikawa, J. *ACS Catal.,* **2015,** *5,* 5947–5950.

5. Fujita, T.; Takazawa, M.; Sugiyama, K.; Suzuki, N.; Ichikawa, J. *Org. Lett.,* **2017,** *19,* 588–591.

6. Fujita, T.; Takahashi I.; Hayashi, M.; Wang, J.; Fuchibe, K.; Ichikawa, J. *Eur. J. Org. Chem.,* **2017,** 262–265.

## 9. $^{1}H$, $^{13}C$, and $^{19}F$ NMR charts

## 9-(Difluoroiodomethyl)-9-phenyl-9,10-dihydrophenanthrene (2a)

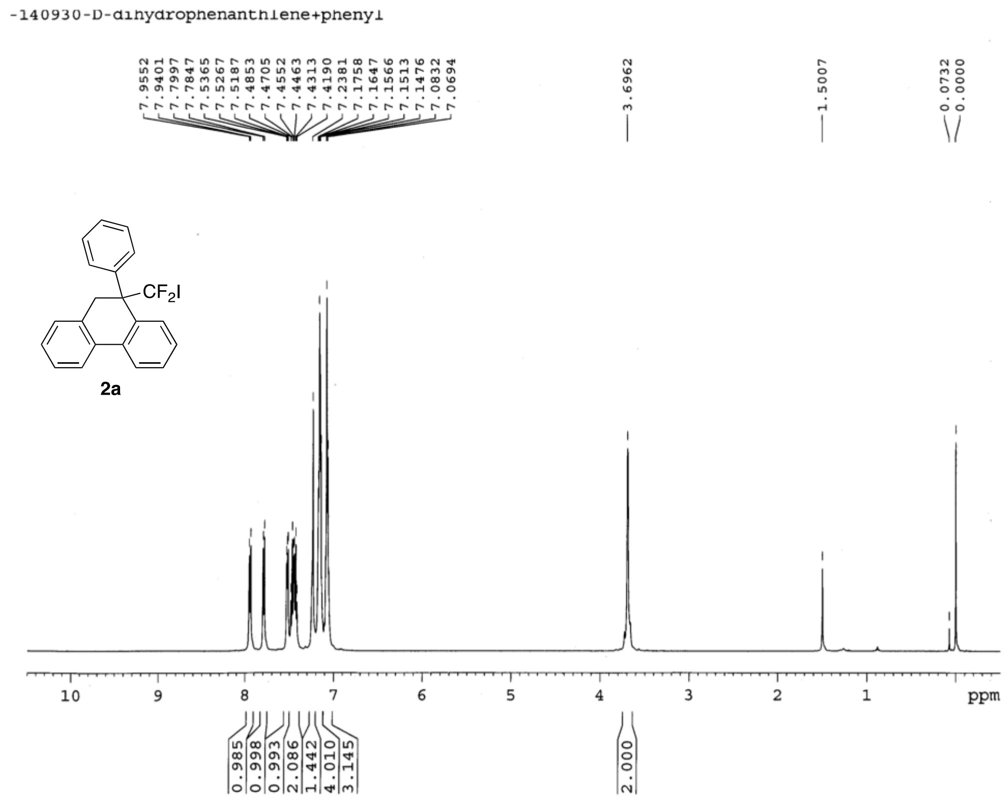

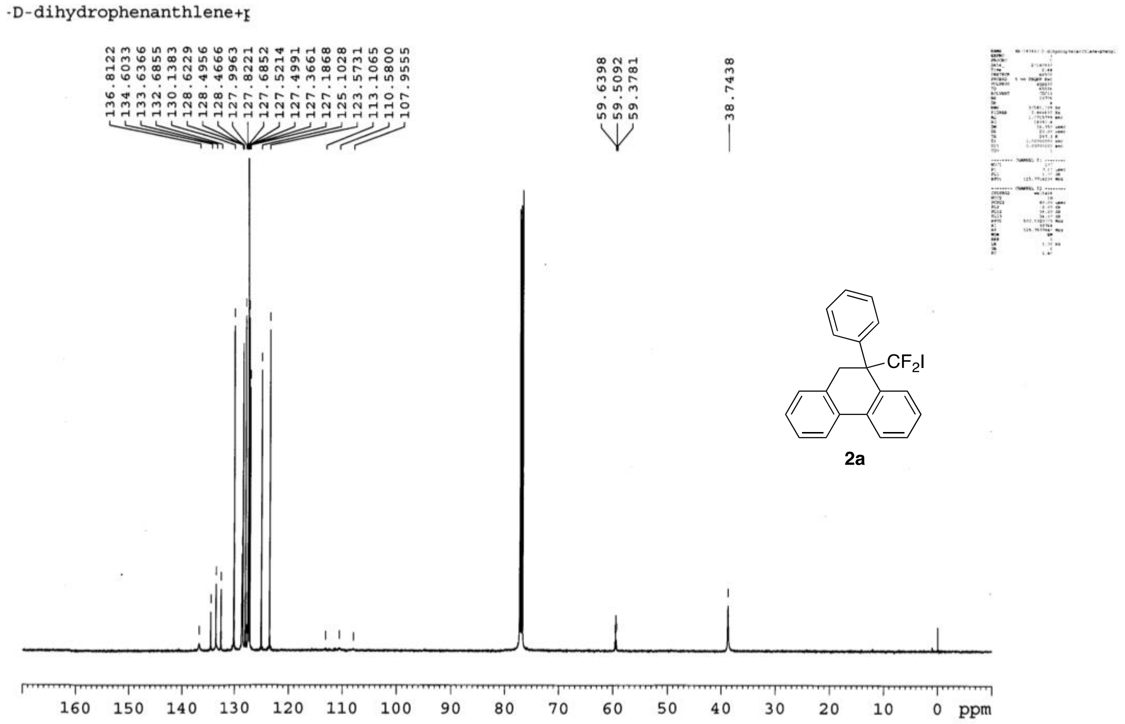

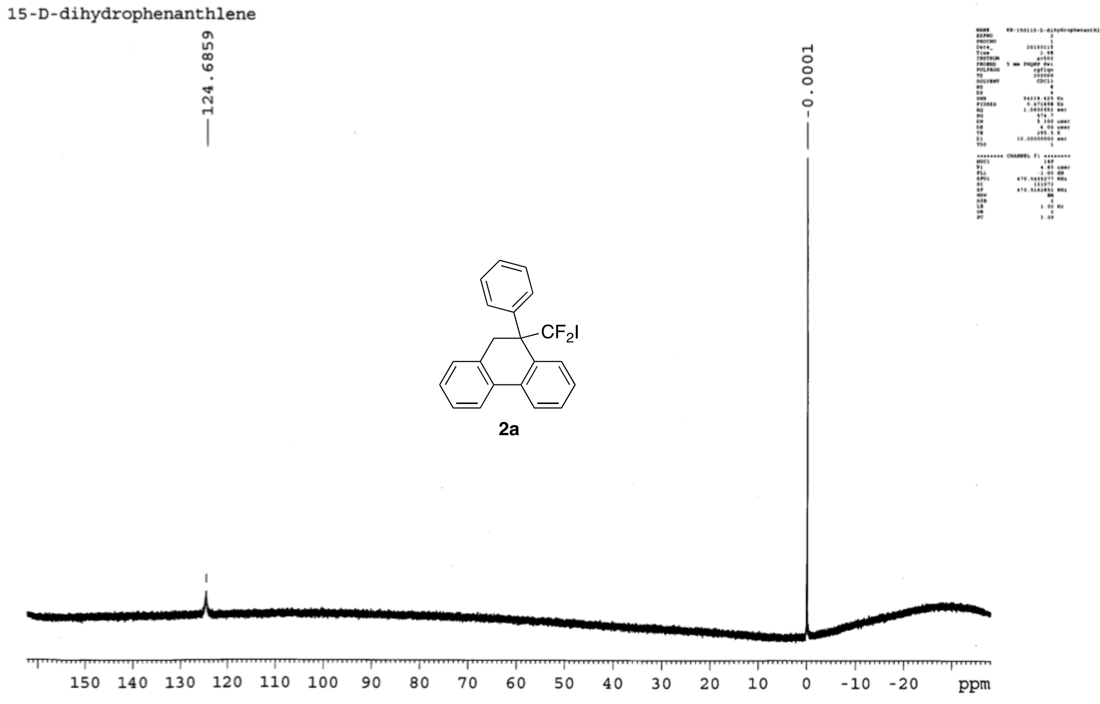


## 9-(Biphenyl-4-yl)-9-(difluoroiodomethyl)-9,10-dihydrophenanthrene (2b)

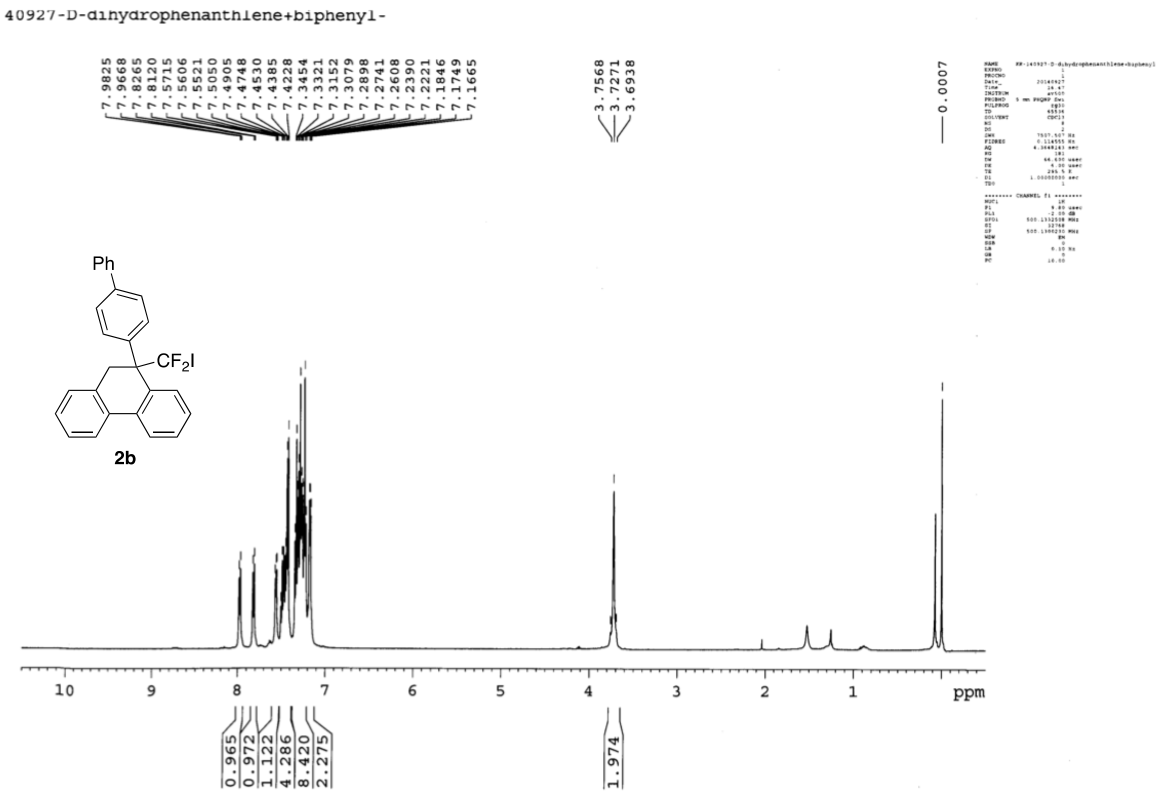

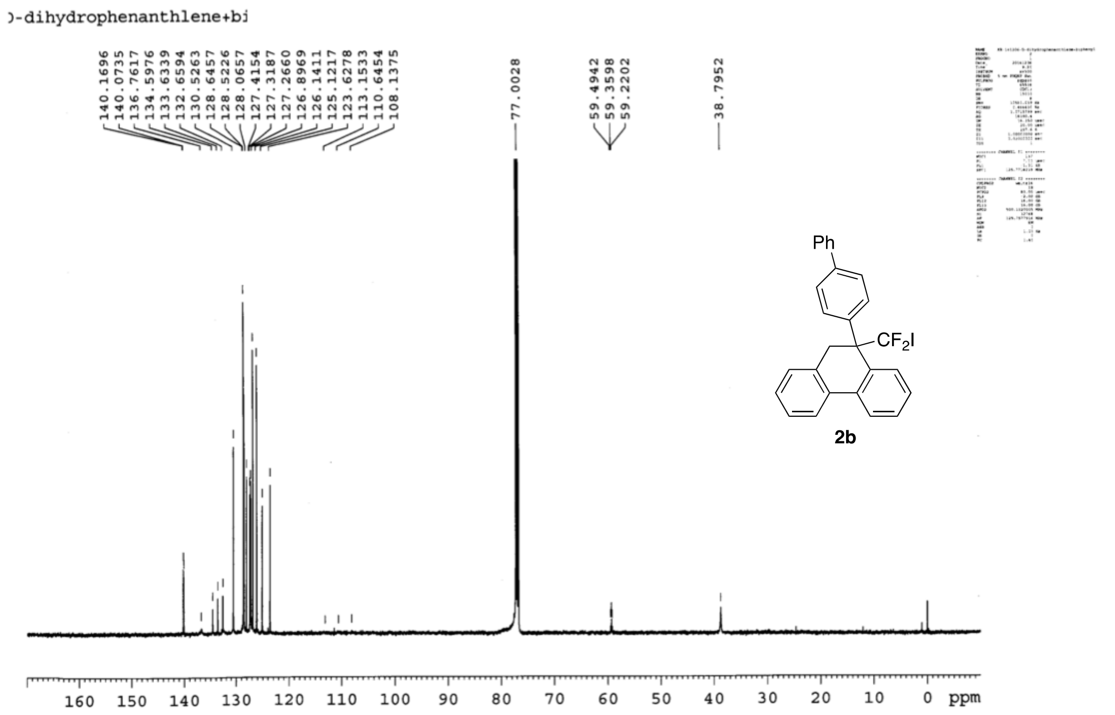
D-dihydrophenanthlene+bi
140.1696
140.0735
136.7617
134.5976
133.6339
132.6594
130.5263
128.6457
128.5226
128.0657
127.4154
127.3187
127.2660
126.8969
126.1411
125.1217
123.6278
113.1533
110.6454
108.1375
77.0028
59.4942
59.3598
59.2202
38.7952
Ph
CF2I
2b
160 150 140 130 120 110 100 90 80 70 60 50 40 30 20 10 0 ppm

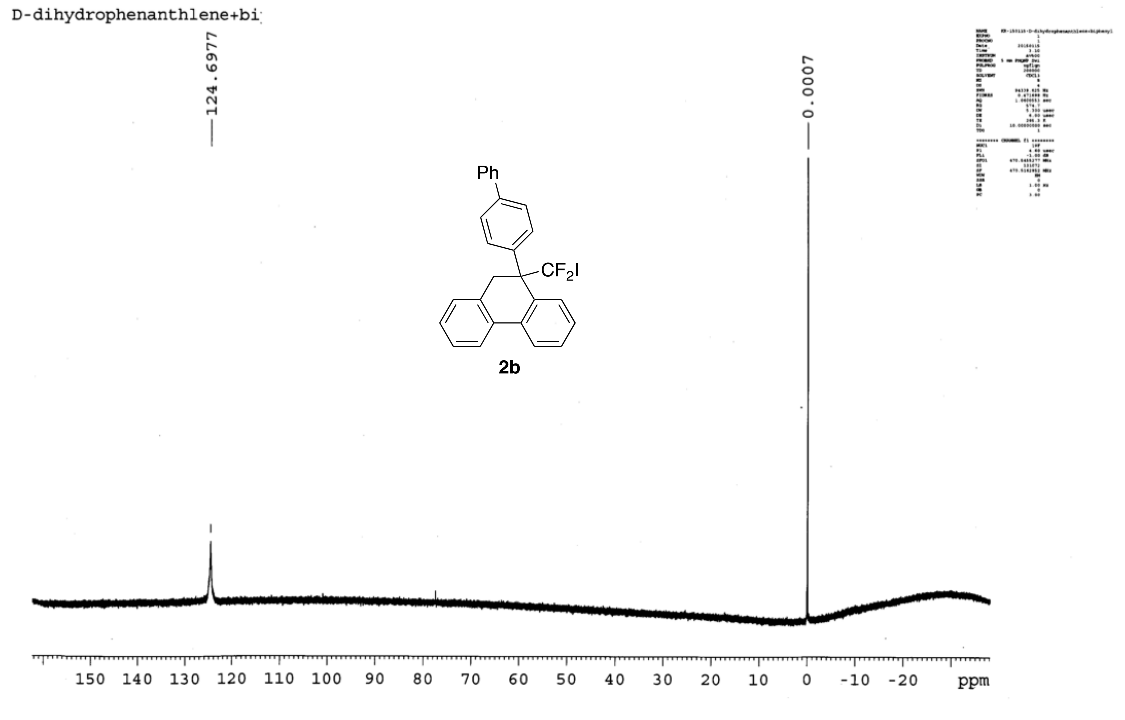
D-dihydrophenanthlene+bi
124.6977
0.0007
Ph
CF2I
2b
150 140 130 120 110 100 90 80 70 60 50 40 30 20 10 0 -10 -20 ppm

**9-(Difluoroiodomethyl)-9-(4-methylphenyl)-9,10-dihydrophenanthrene (2c)**

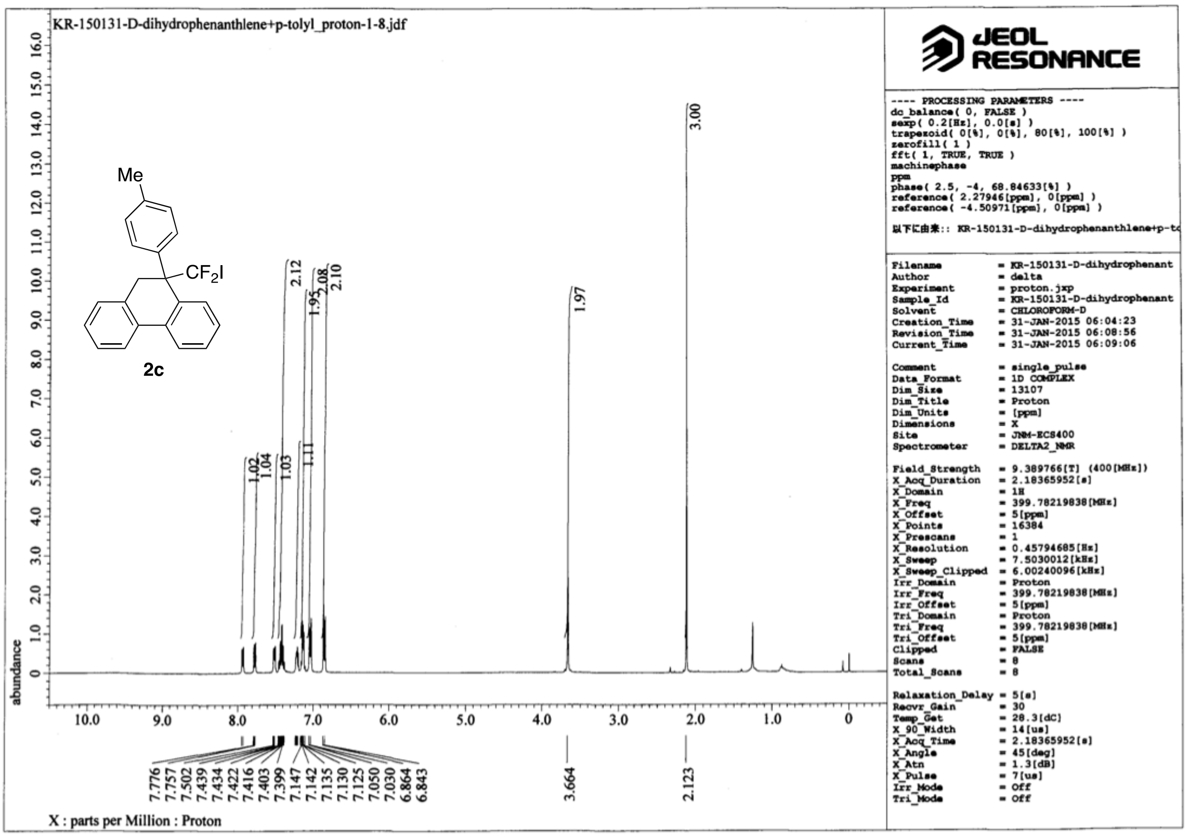

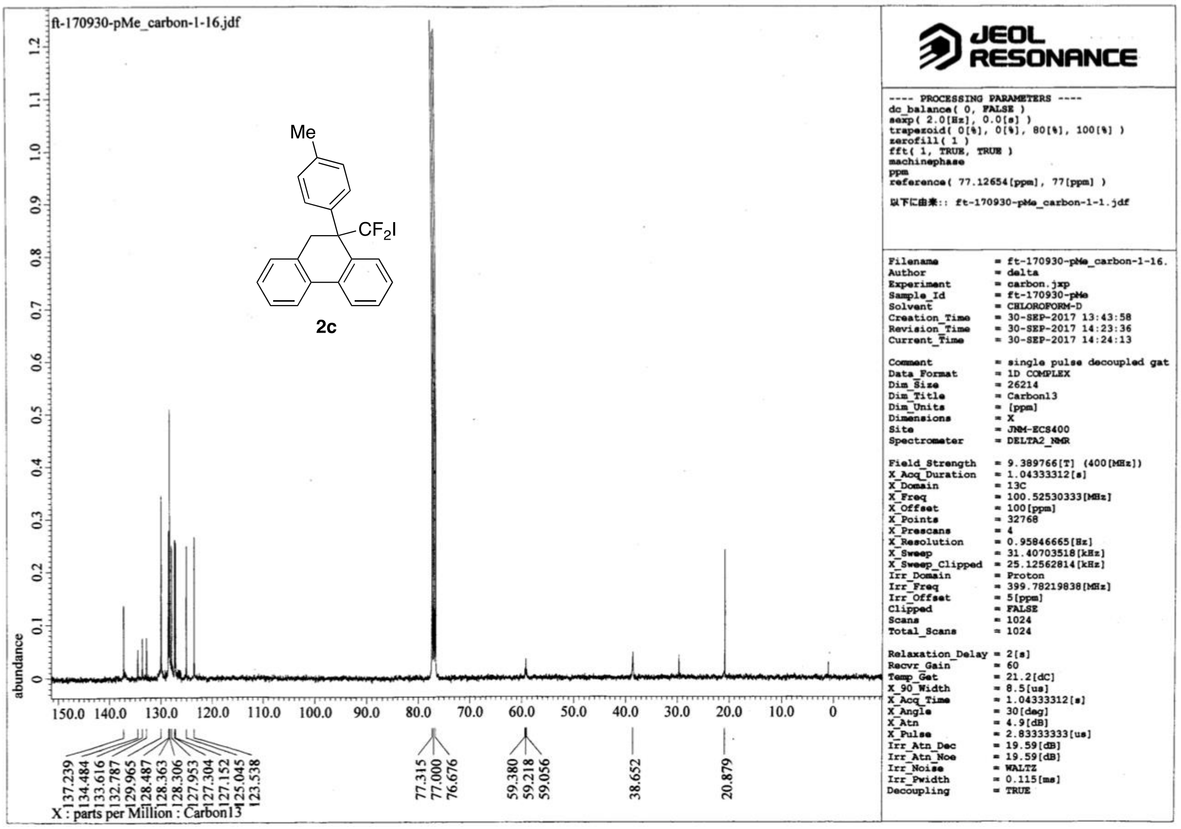

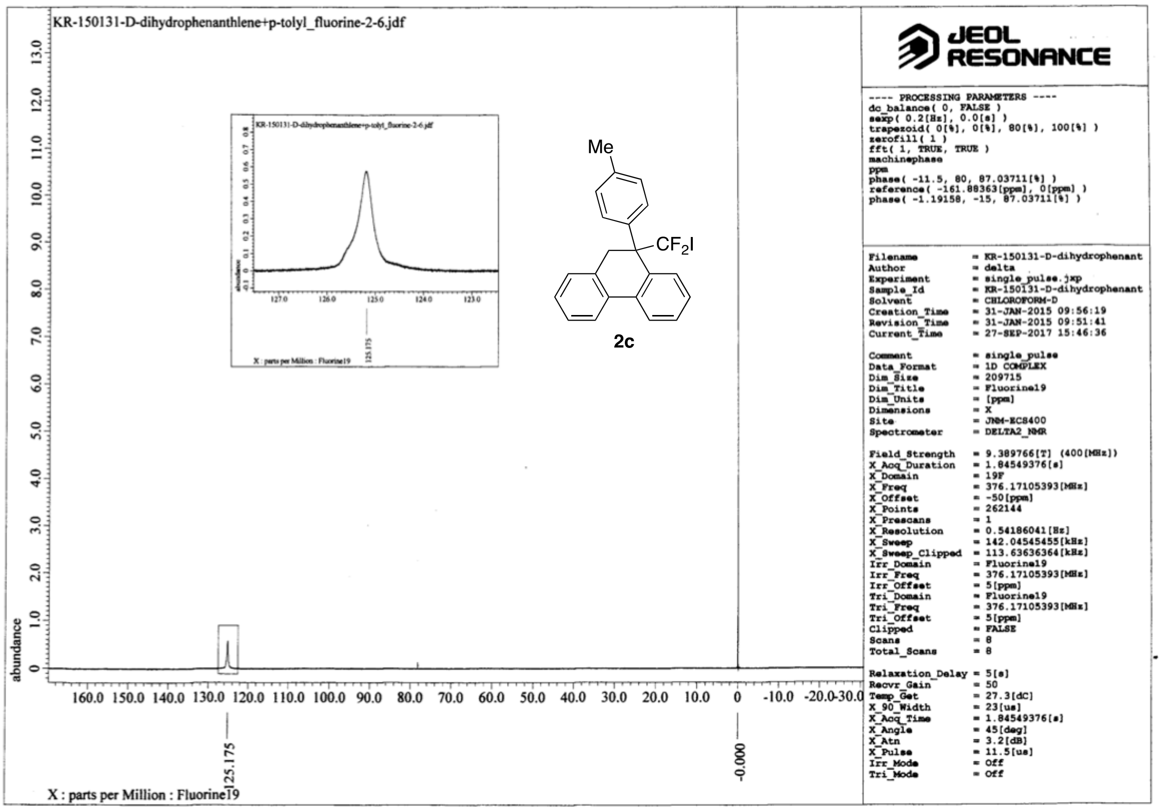


## 9-(Difluoroiodomethyl)-9-(3-methylphenyl)-9,10-dihydrophenanthrene (2d)

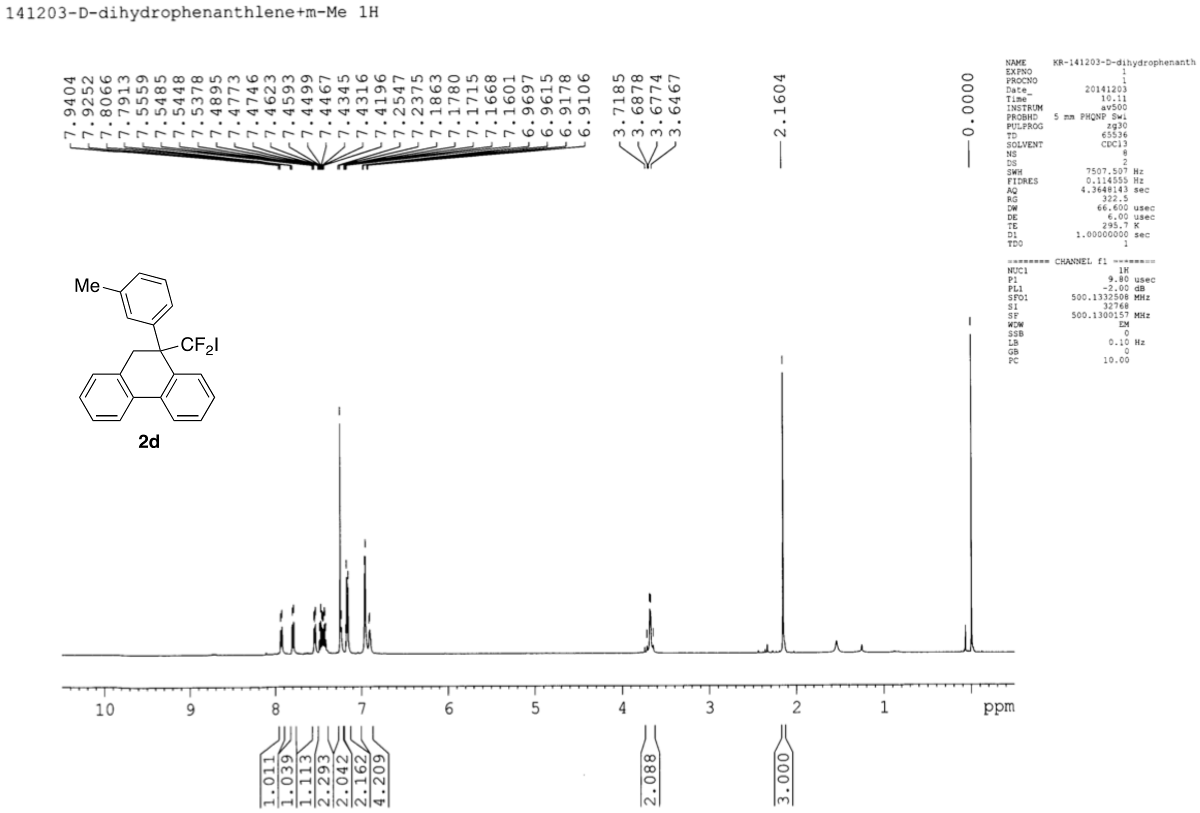

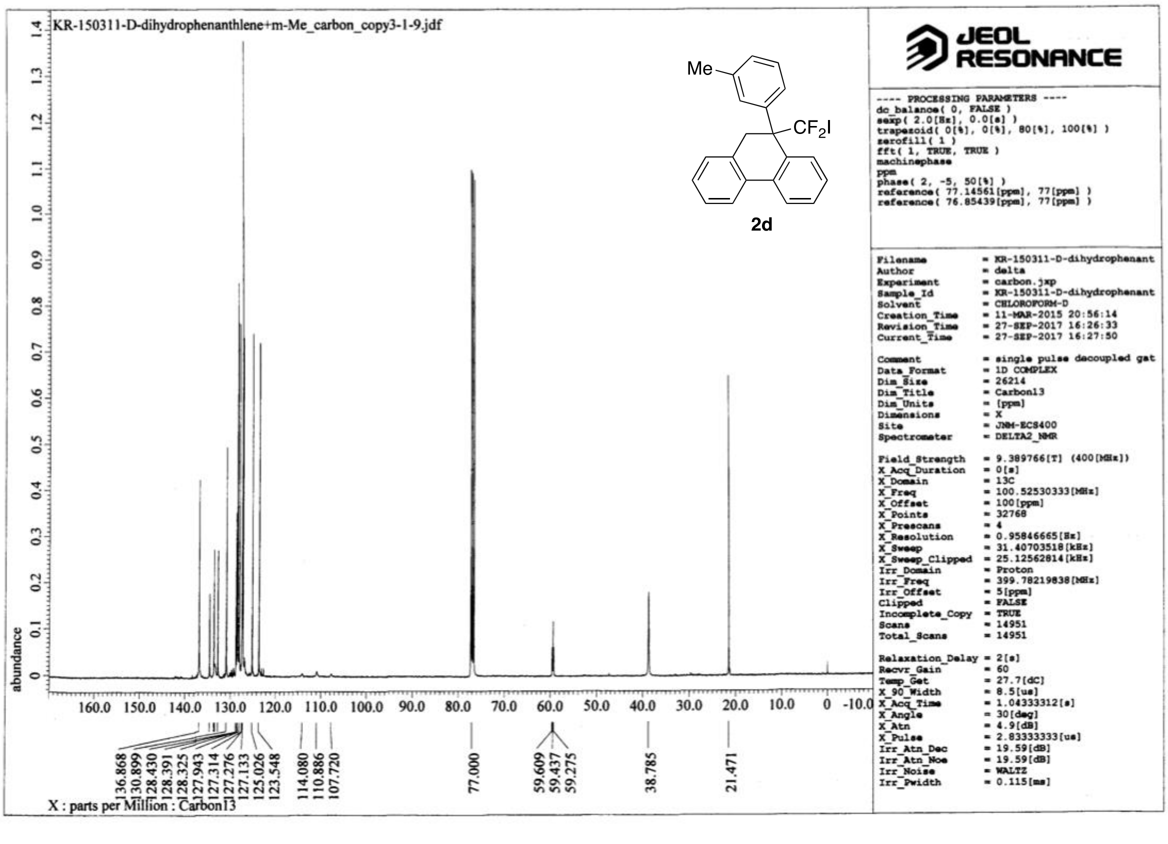
KR-150311-D-dihydrophenanthlene+m-Me_carbon_copy3-1-9.jdf
2d
136.868
130.899
128.430
128.391
128.325
127.943
127.314
127.276
127.133
125.026
123.548
114.080
110.886
107.720
77.000
59.609
59.437
59.275
38.785
21.471
X : parts per Million : Carbon13
abundance

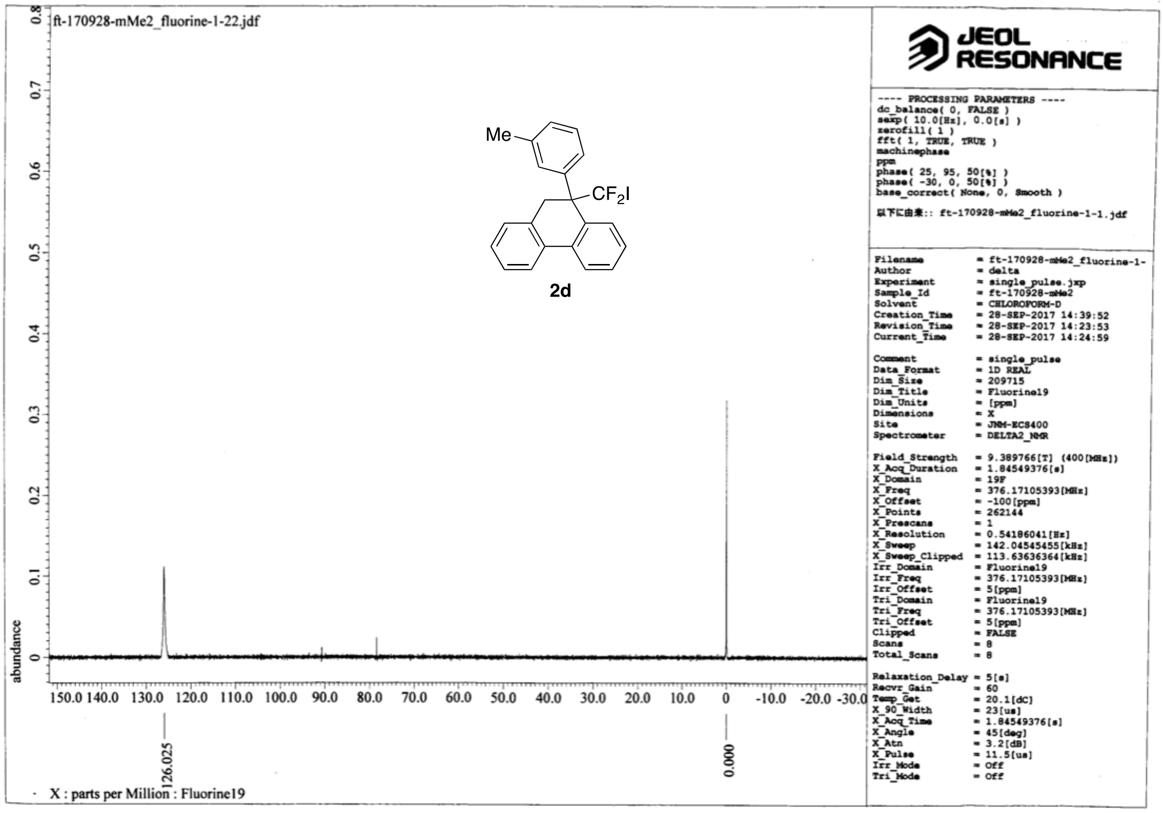
ft-170928_mMe2_fluorine-1-22.jdf
2d
126.025
0.000
X : parts per Million : Fluorine19
abundance

## 9-(Difluoroiodomethyl)-9-(4-methoxyphenyl)-9,10-dihydrophenanthrene (2e)

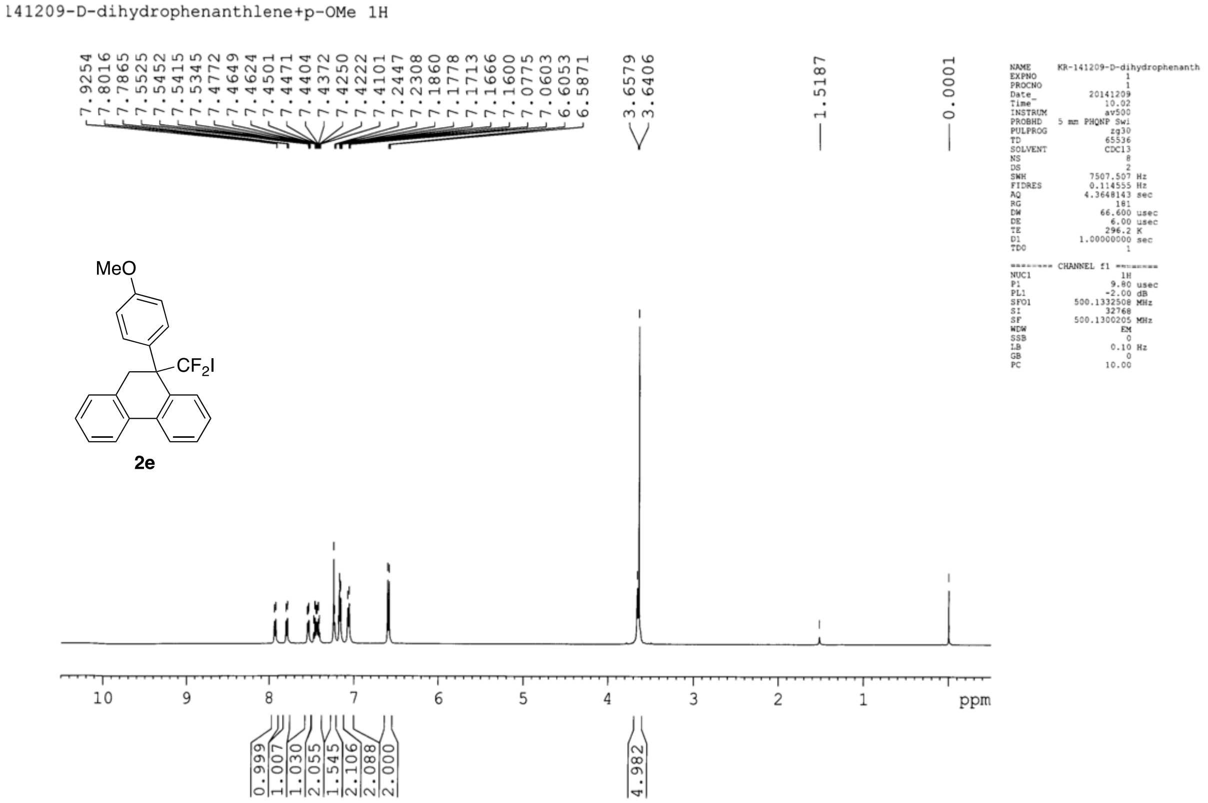


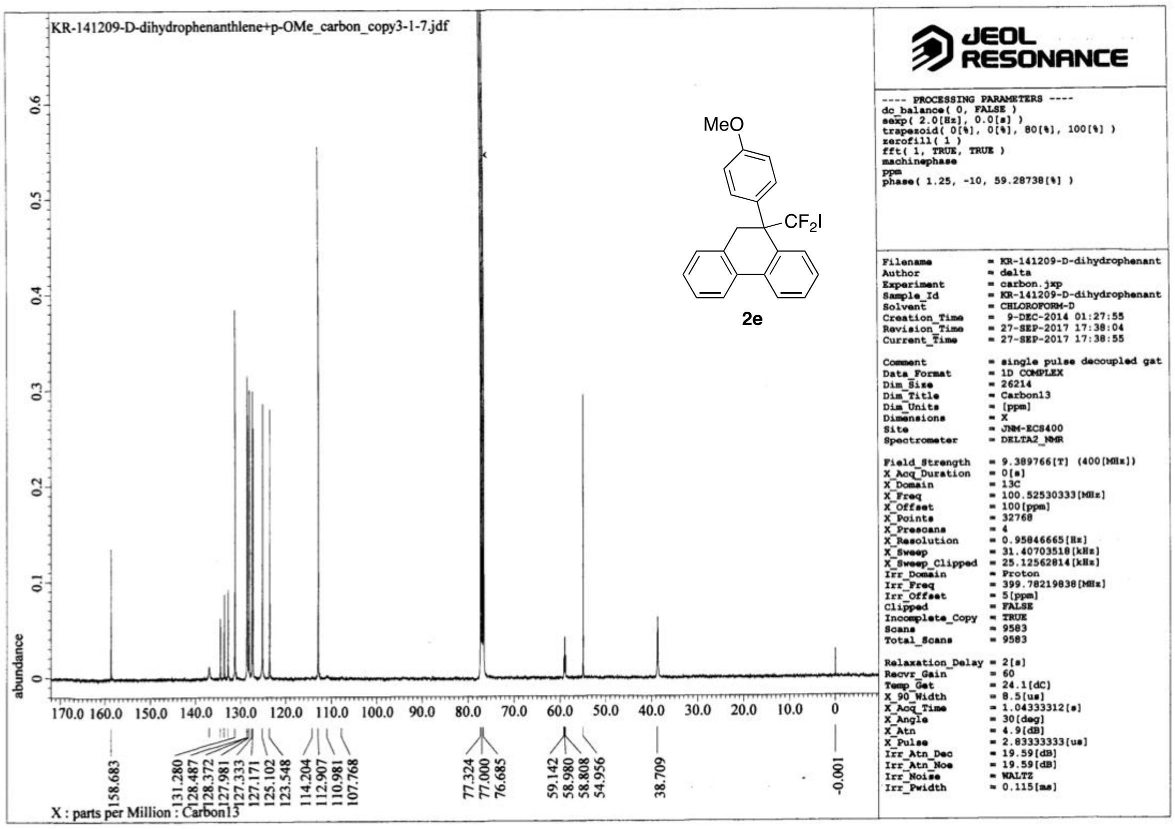

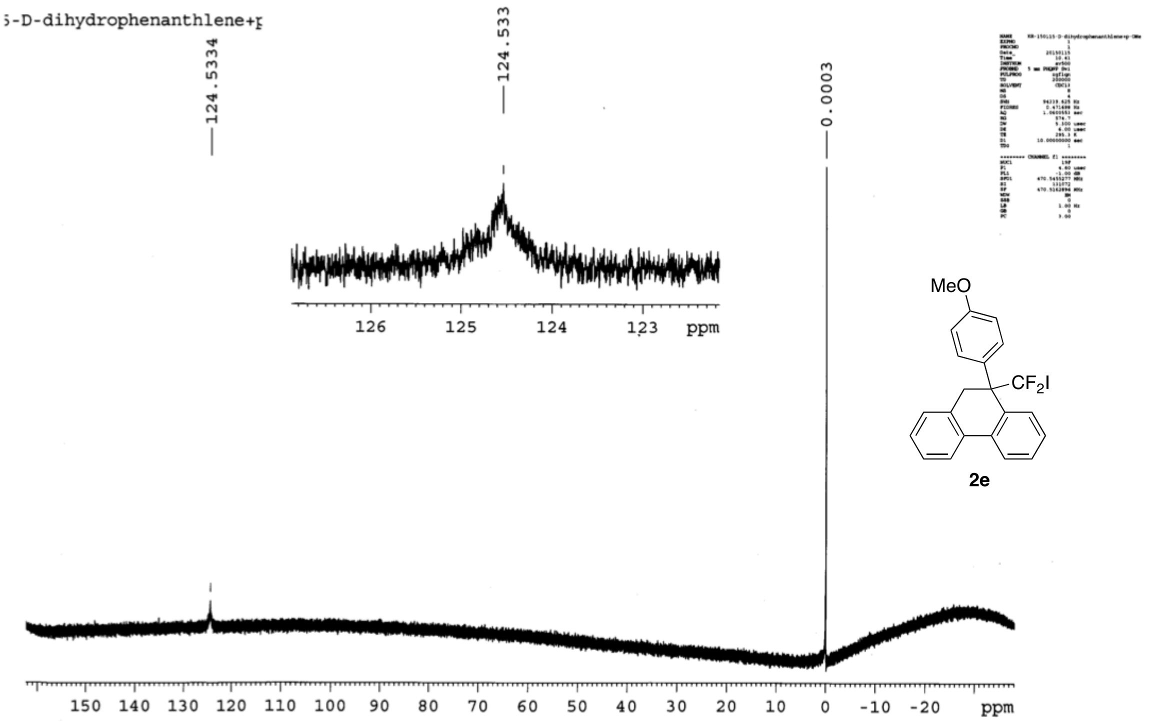


## 10-(Difluoroiodomethyl)-2-methyl-10-phenyl-9,10-dihydrophenanthrene (2f)

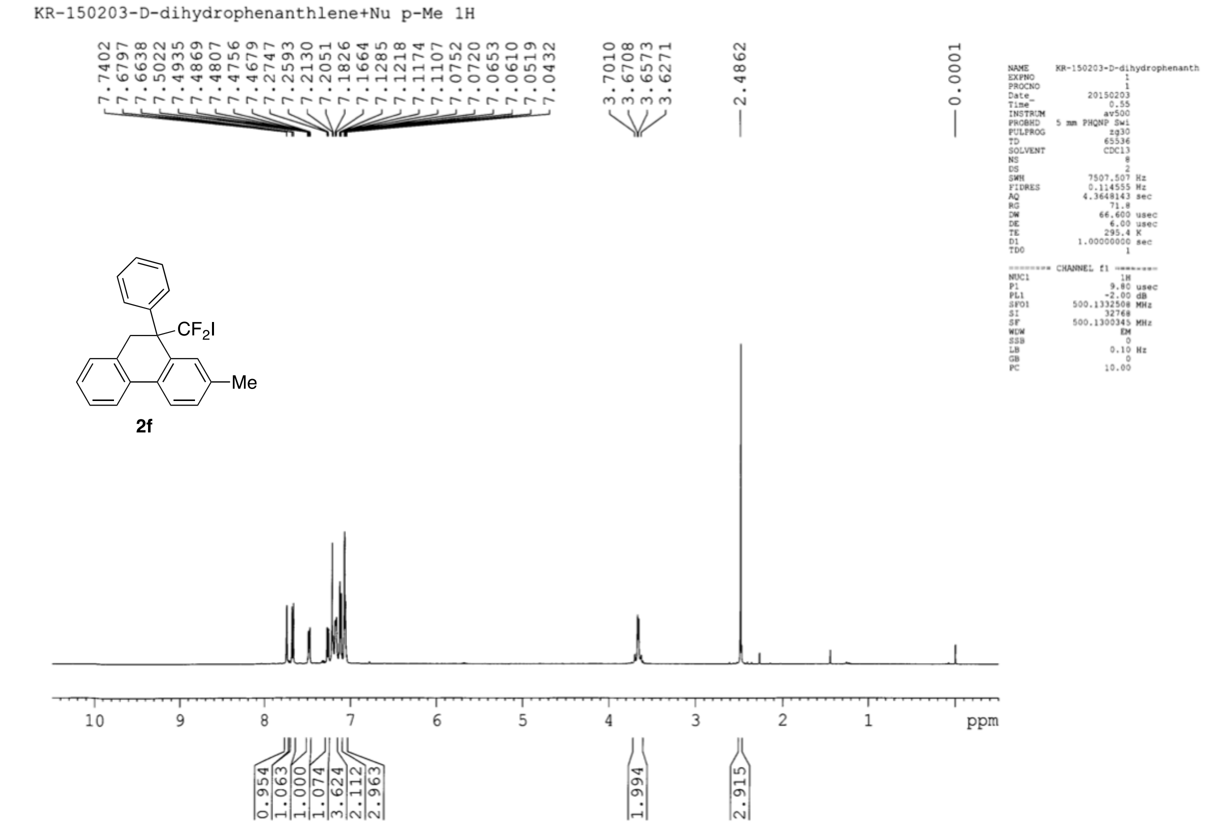

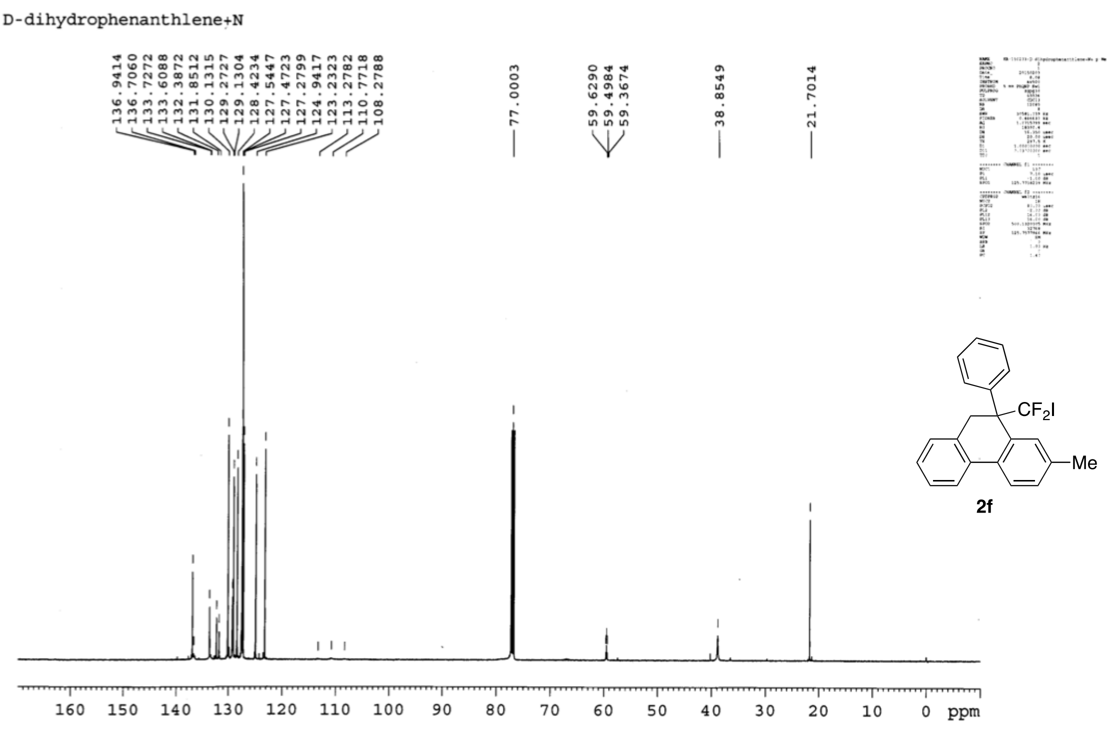
D-dihydrophenanthlene+N
136.9414
136.7060
133.7272
133.6088
132.3872
131.8512
130.1315
129.2727
129.1304
128.4234
127.5447
127.4723
127.2799
124.9417
123.2323
113.2782
110.7718
108.2788
77.0003
59.6290
59.4984
59.3674
38.8549
21.7014
$CF_2I$
Me
2f
160 150 140 130 120 110 100 90 80 70 60 50 40 30 20 10 0 ppm

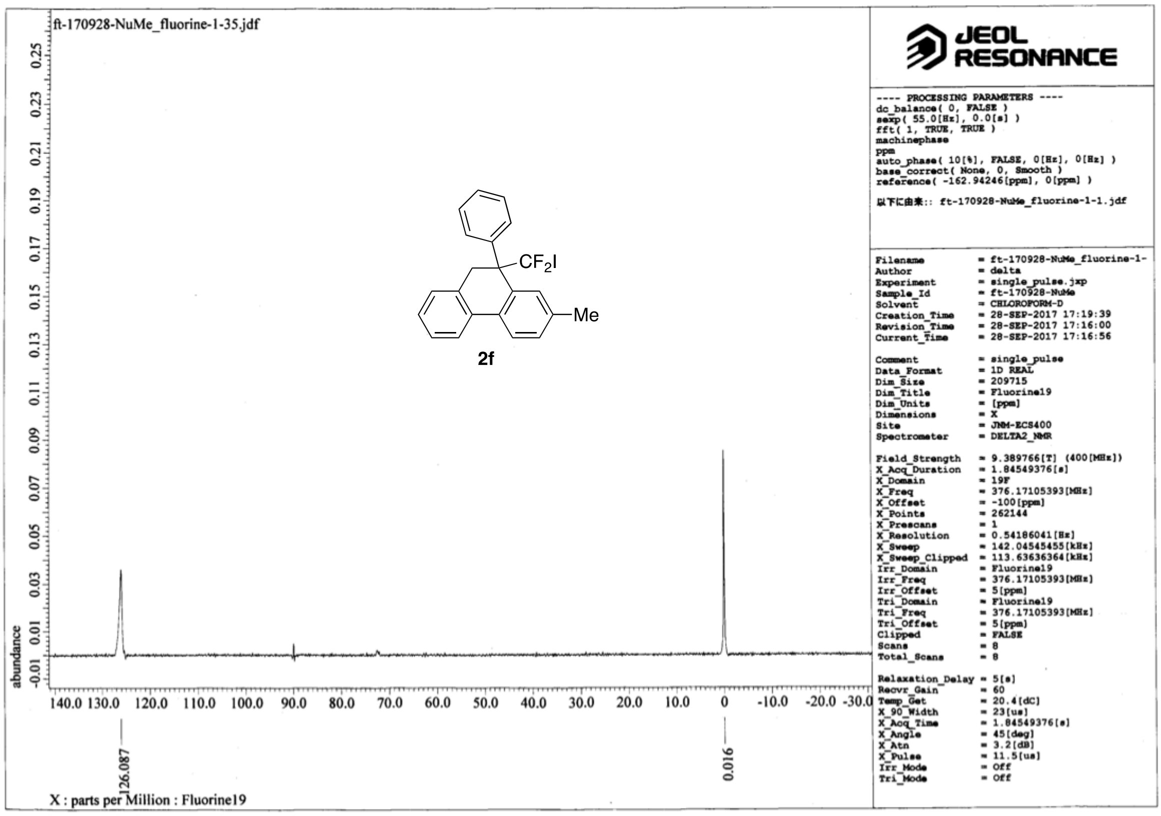
ft-170928-NuMe_fluorine-1-35.jdf
JEOL RESONANCE
---- PROCESSING PARAMETERS ----
dc_balance( 0, FALSE )
sexp( 55.0[Hz], 0.0[s] )
fft( 1, TRUE, TRUE )
machinephase
ppm
auto_phase( 10[%], FALSE, 0[Hz], 0[Hz] )
base_correct( None, 0, Smooth )
reference( -162.94246[ppm], 0[ppm] )
以下に由来:: ft-170928-NuMe_fluorine-1-1.jdf
Filename = ft-170928-NuMe_fluorine-1-
Author = delta
Experiment = single_pulse.jxp
Sample_Id = ft-170928-NuMe
Solvent = CHLOROFORM-D
Creation_Time = 28-SEP-2017 17:19:39
Revision_Time = 28-SEP-2017 17:16:00
Current_Time = 28-SEP-2017 17:16:56
Comment = single_pulse
Data_Format = 1D REAL
Dim_Size = 209715
Dim_Title = Fluorine19
Dim_Units = [ppm]
Dimensions = X
Site = JNM-ECS400
Spectrometer = DELTA2_NMR
Field_Strength = 9.389766[T] (400[MHz])
X_Acq_Duration = 1.84549376[s]
X_Domain = 19F
X_Freq = 376.17105393[MHz]
X_Offset = -100[ppm]
X_Points = 262144
X_Prescans = 1
X_Resolution = 0.54186041[Hz]
X_Sweep = 142.04545455[kHz]
X_Sweep_Clipped = 113.63636364[kHz]
Irr_Domain = Fluorine19
Irr_Freq = 376.17105393[MHz]
Irr_Offset = 5[ppm]
Tri_Domain = Fluorine19
Tri_Freq = 376.17105393[MHz]
Tri_Offset = 5[ppm]
Clipped = FALSE
Scans = 8
Total_Scans = 8
Relaxation_Delay = 5[s]
Recvr_Gain = 60
Temp_Get = 20.4[dC]
X_90_Width = 23[us]
X_Acq_Time = 1.84549376[s]
X_Angle = 45[deg]
X_Atn = 3.2[dB]
X_Pulse = 11.5[us]
Irr_Mode = Off
Tri_Mode = Off
$CF_2I$
Me
2f
abundance
0.25 0.23 0.21 0.19 0.17 0.15 0.13 0.11 0.09 0.07 0.05 0.03 0.01 -0.01
140.0 130.0 120.0 110.0 100.0 90.0 80.0 70.0 60.0 50.0 40.0 30.0 20.0 10.0 0 -10.0 -20.0 -30.0
126.087
0.016
X : parts per Million : Fluorine19

## 10-(Difluoroiodomethyl)-2-methoxy-10-phenyl-9,10-dihydrophenanthrene (2g)

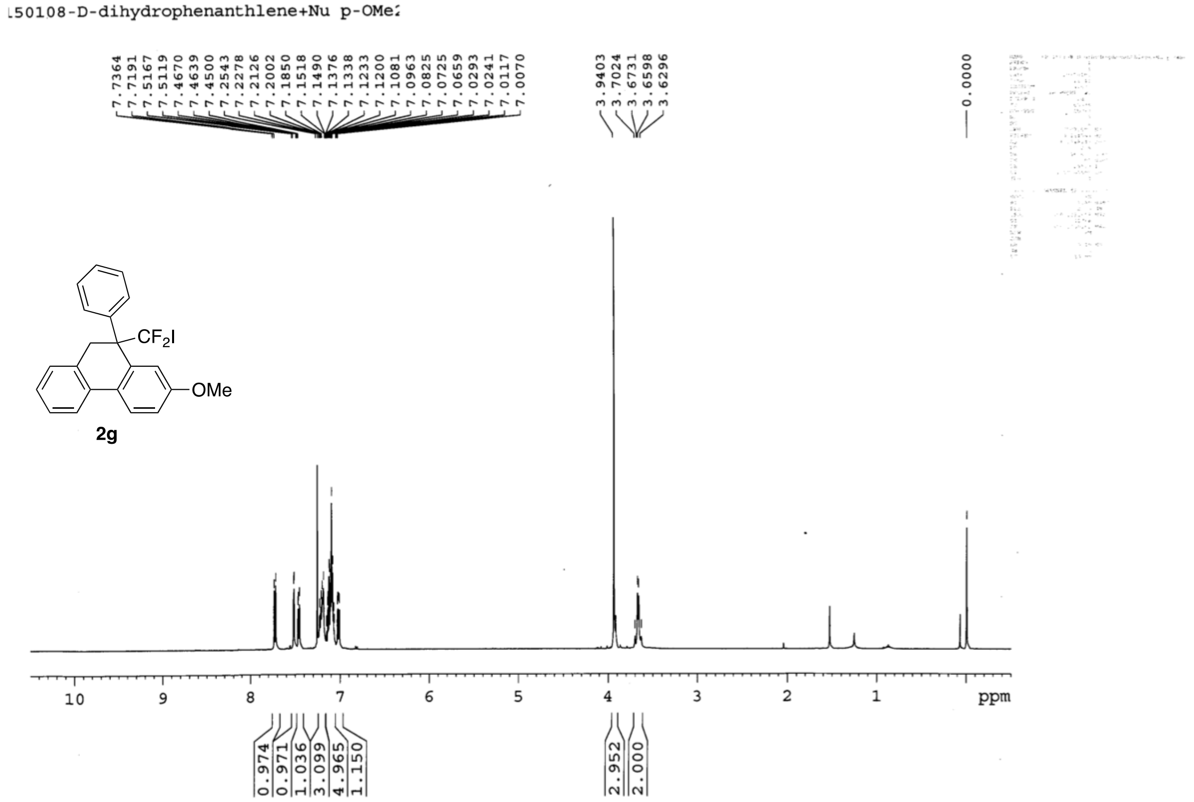


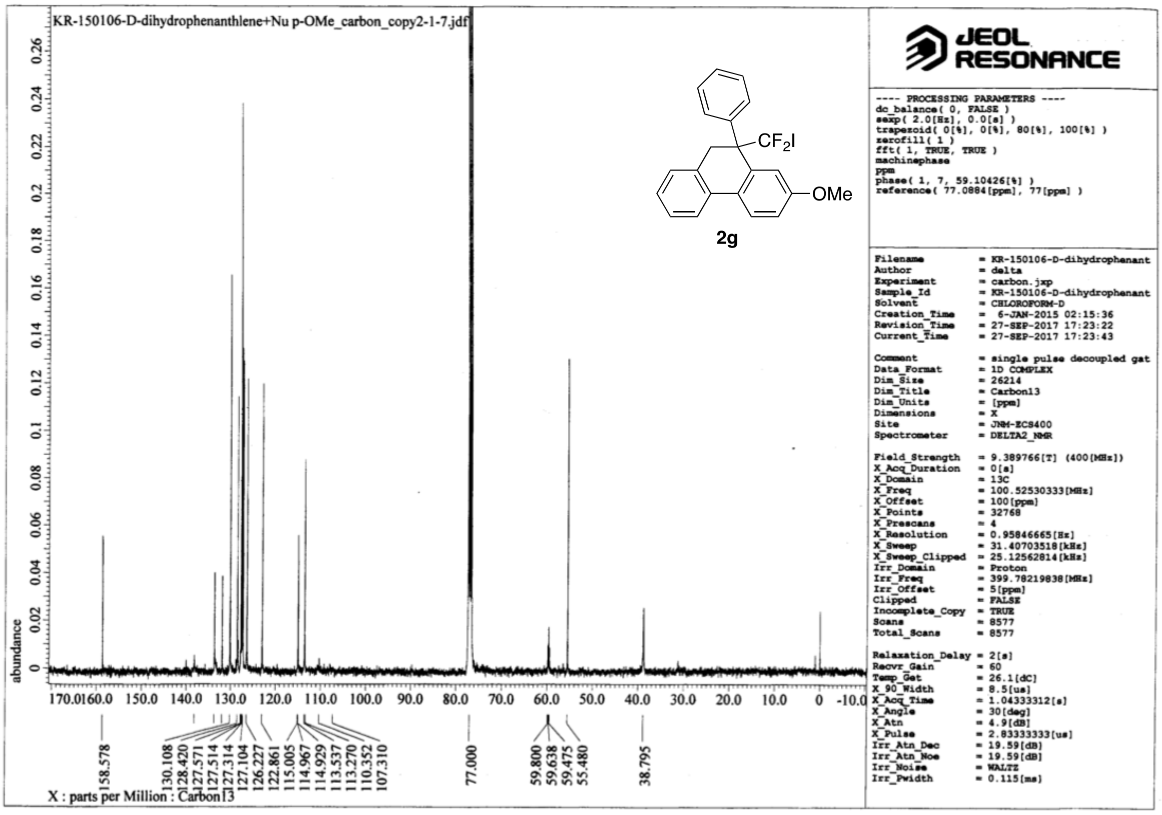

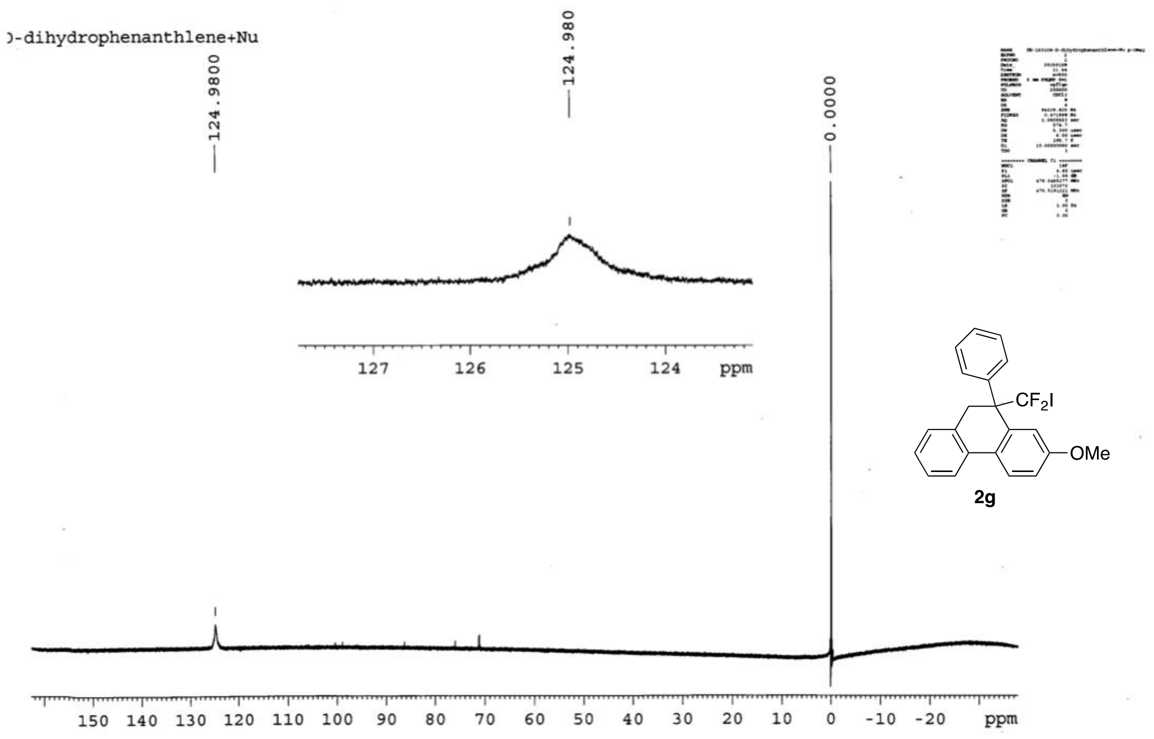


## 9-(Difluoromethyl)-9-phenyl-9,10-dihydrophenanthrene (4a)

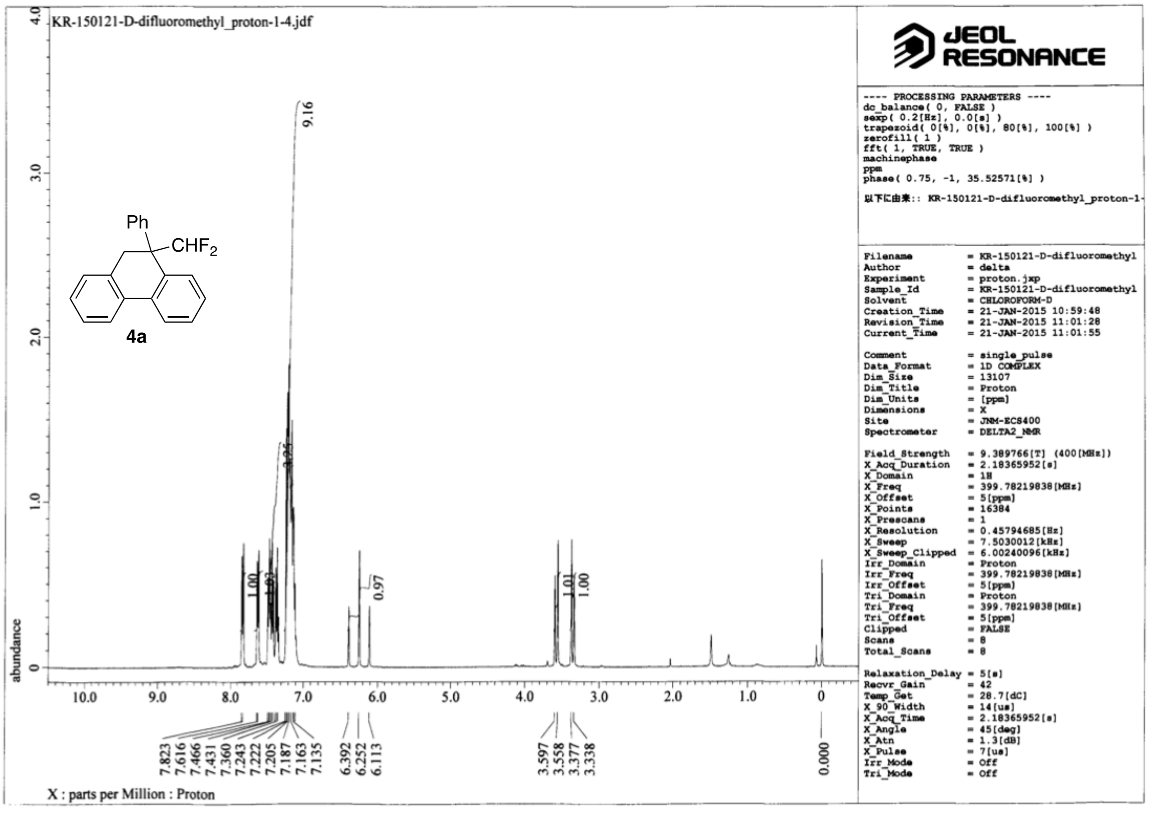

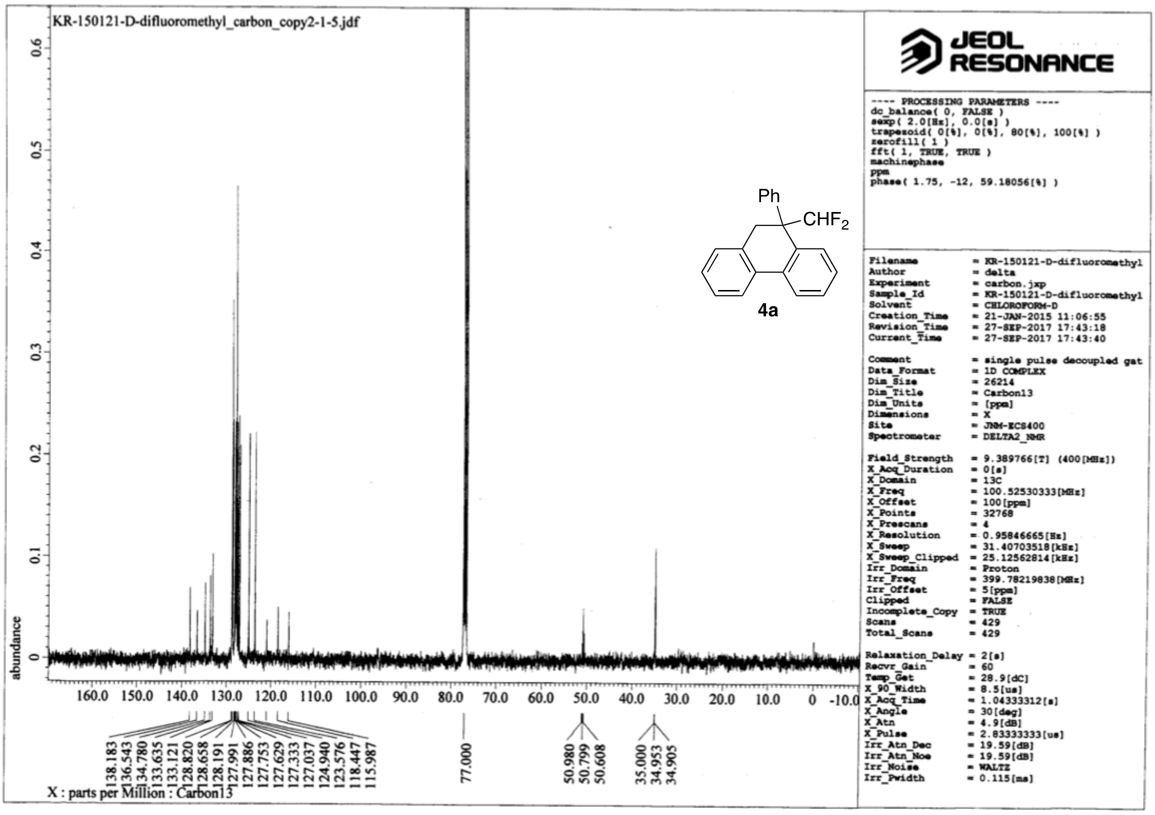
KR-150121-D-difluoromethyl_carbon_copy2-1-5.jdf
JEOL RESONANCE
abundance
X : parts per Million : Carbon13
138.183
136.543
134.780
133.635
133.121
128.820
128.658
128.191
127.991
127.886
127.753
127.629
127.333
127.037
124.940
123.576
118.447
115.987
77.000
50.980
50.799
50.608
35.000
34.953
34.905
Ph
CHF2
4a

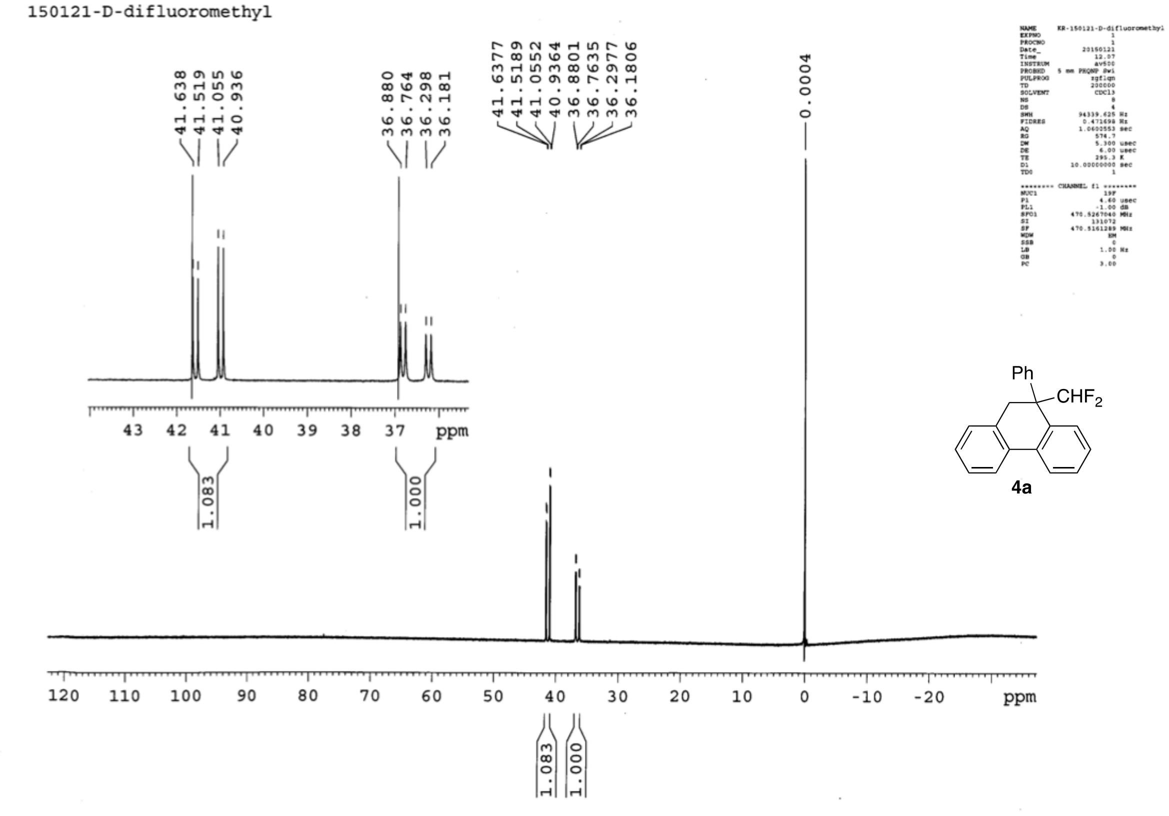
150121-D-difluoromethyl
41.638
41.519
41.055
40.936
36.880
36.764
36.298
36.181
41.6377
41.5189
40.0552
40.9364
36.8801
36.7635
36.2977
36.1806
0.0004
1.083
1.000
ppm
Ph
CHF2
4a

## 5,5-Difluoro-6-iodo-6,7-dihydro-5*H*-dibenzo[*a*,*c*][7]annulene (6a)

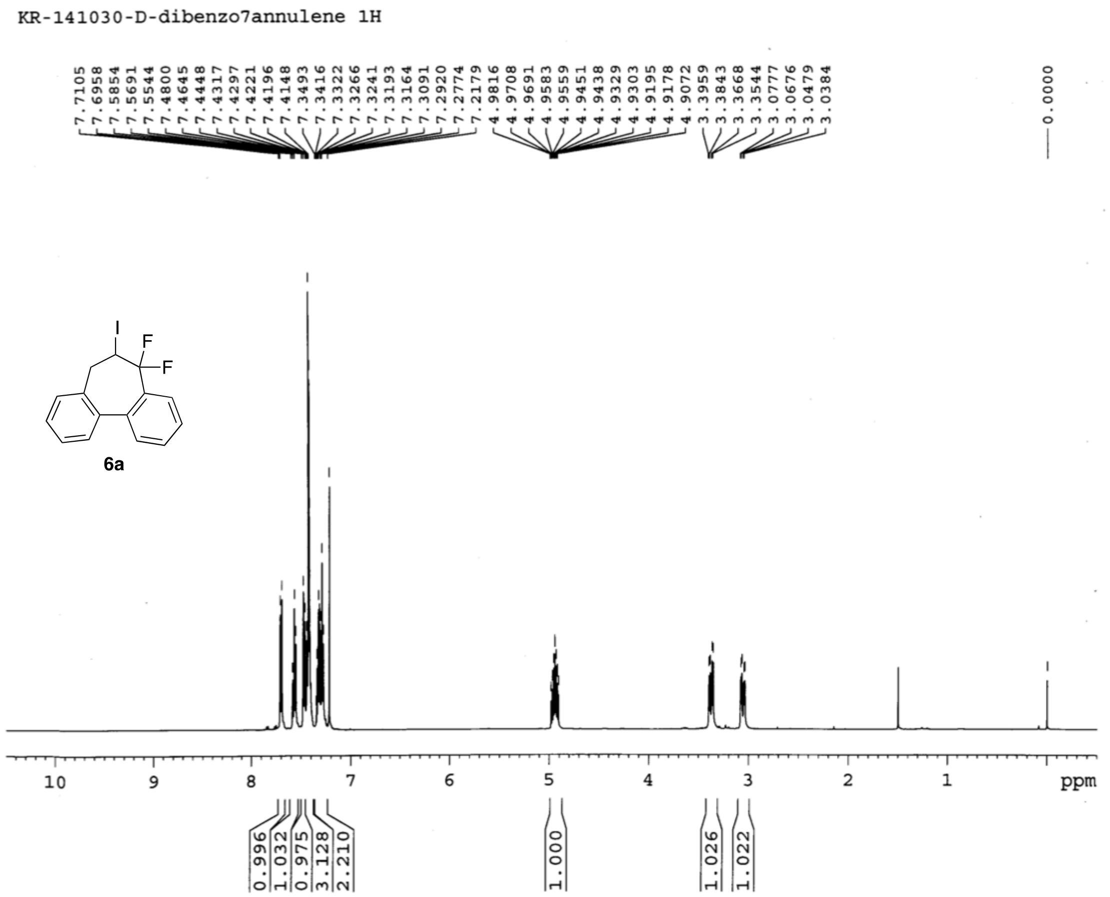


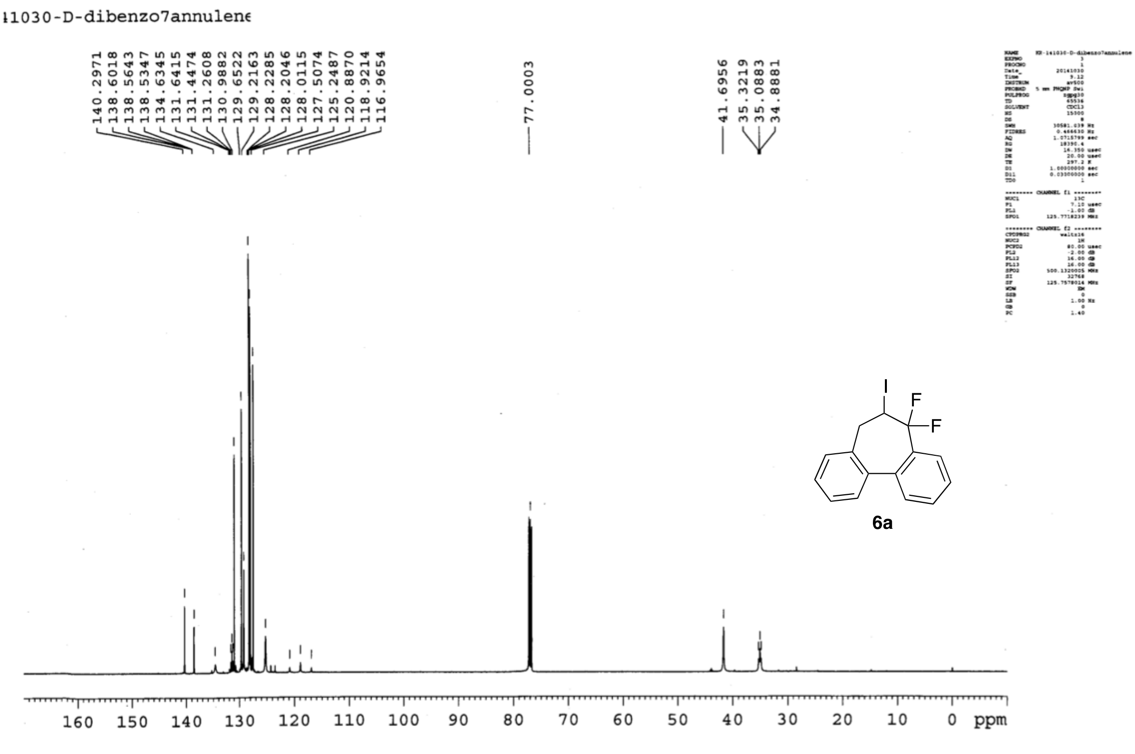

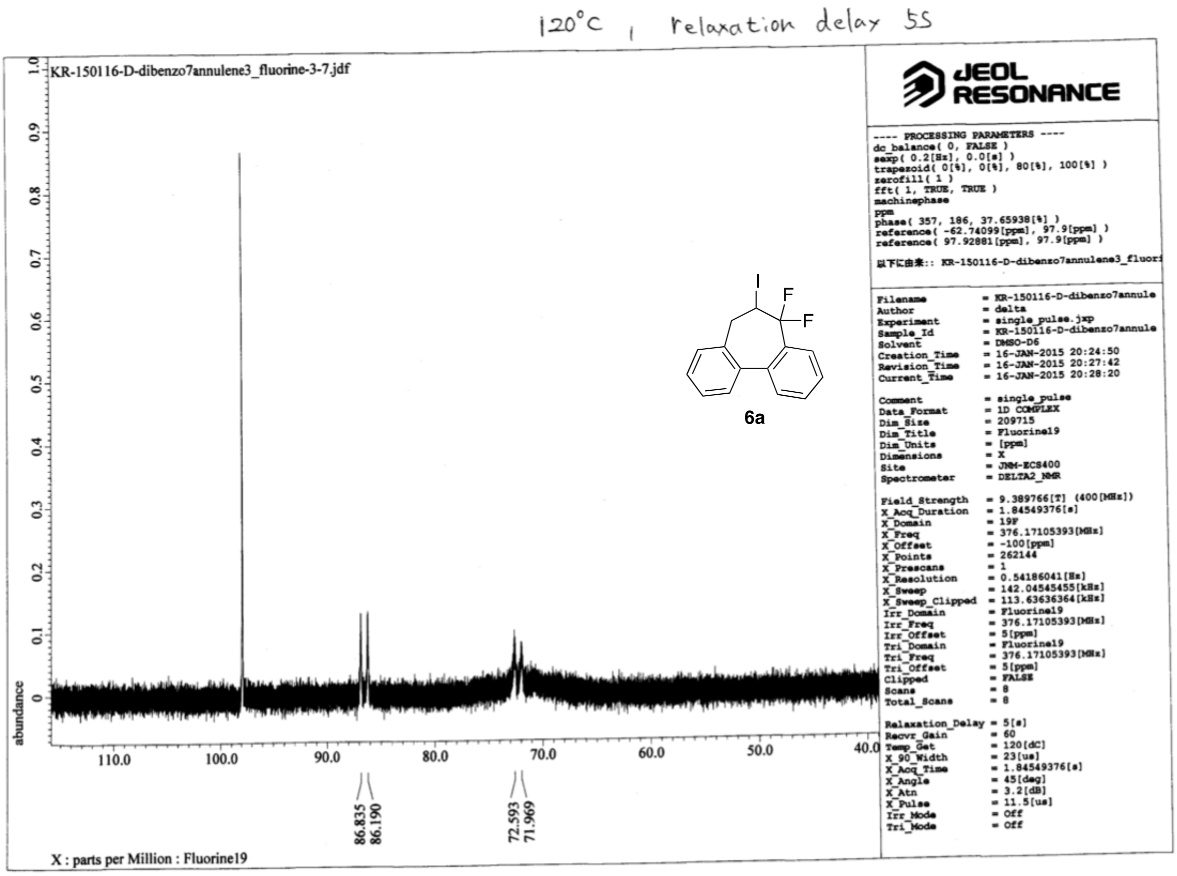


## 5,5-Difluoro-6-iodo-3-methyl-6,7-dihydro-5*H*-dibenzo[*a*,*c*][7]annulene (6b)

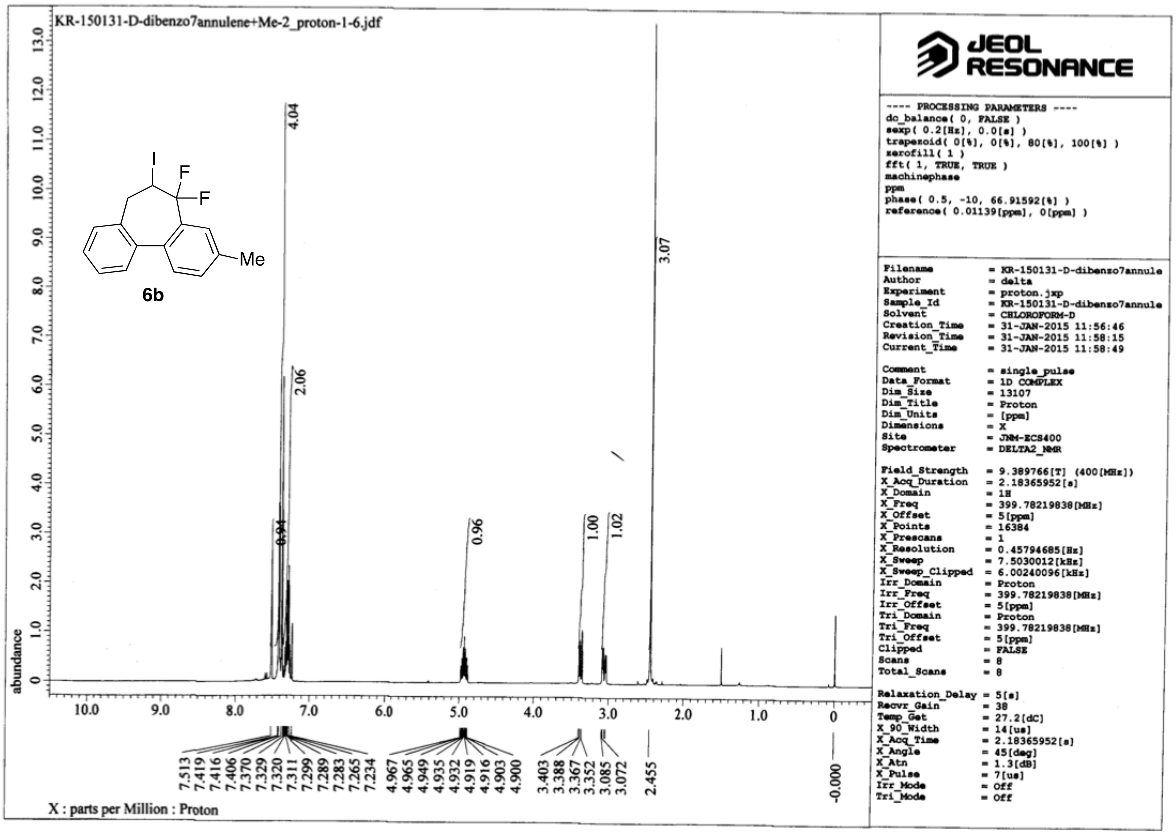

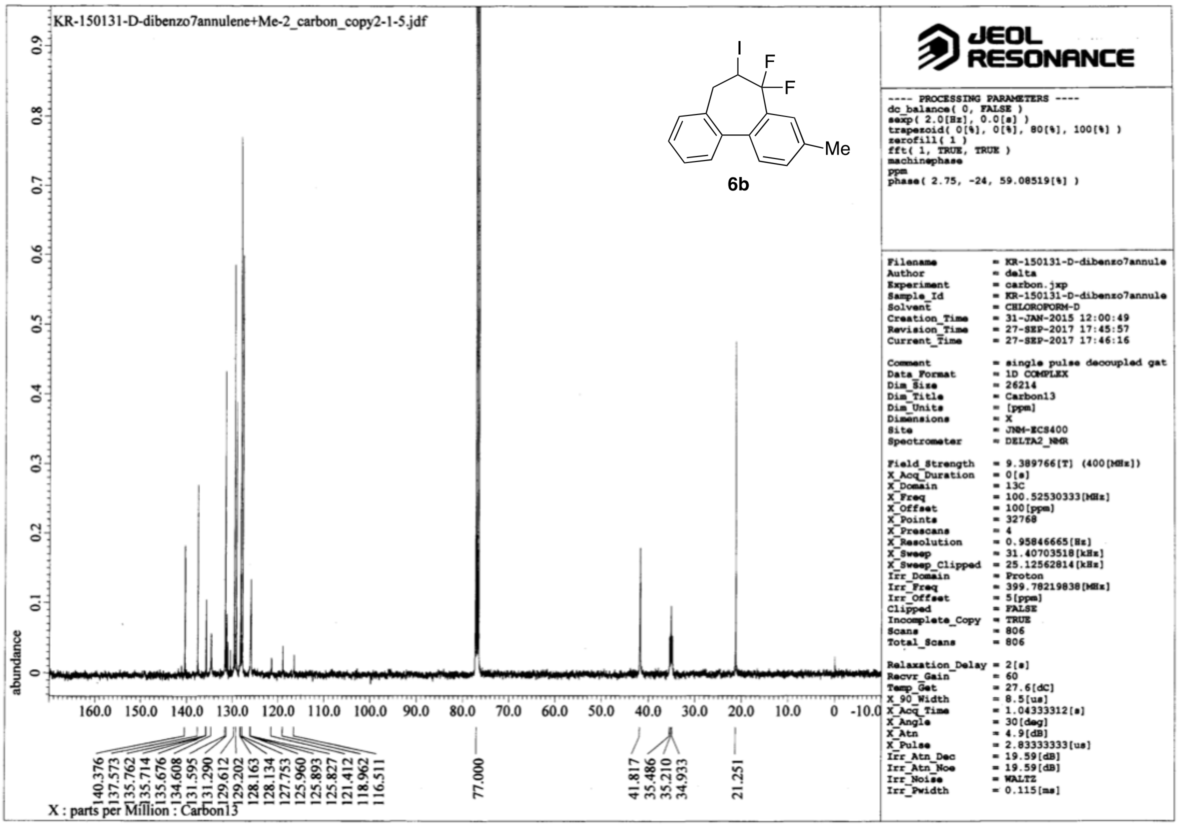
KR-150131-D-dibenzo7annulene+Me-2_carbon_copy2-1-5.jdf
JEOL RESONANCE
I
F
F
Me
6b
abundance
X : parts per Million : Carbon13
140.376
137.573
135.762
135.714
135.676
134.608
131.595
131.290
129.612
129.202
128.163
128.134
127.753
125.960
125.893
125.827
121.412
118.962
116.511
77.000
41.817
35.486
35.210
34.933
21.251

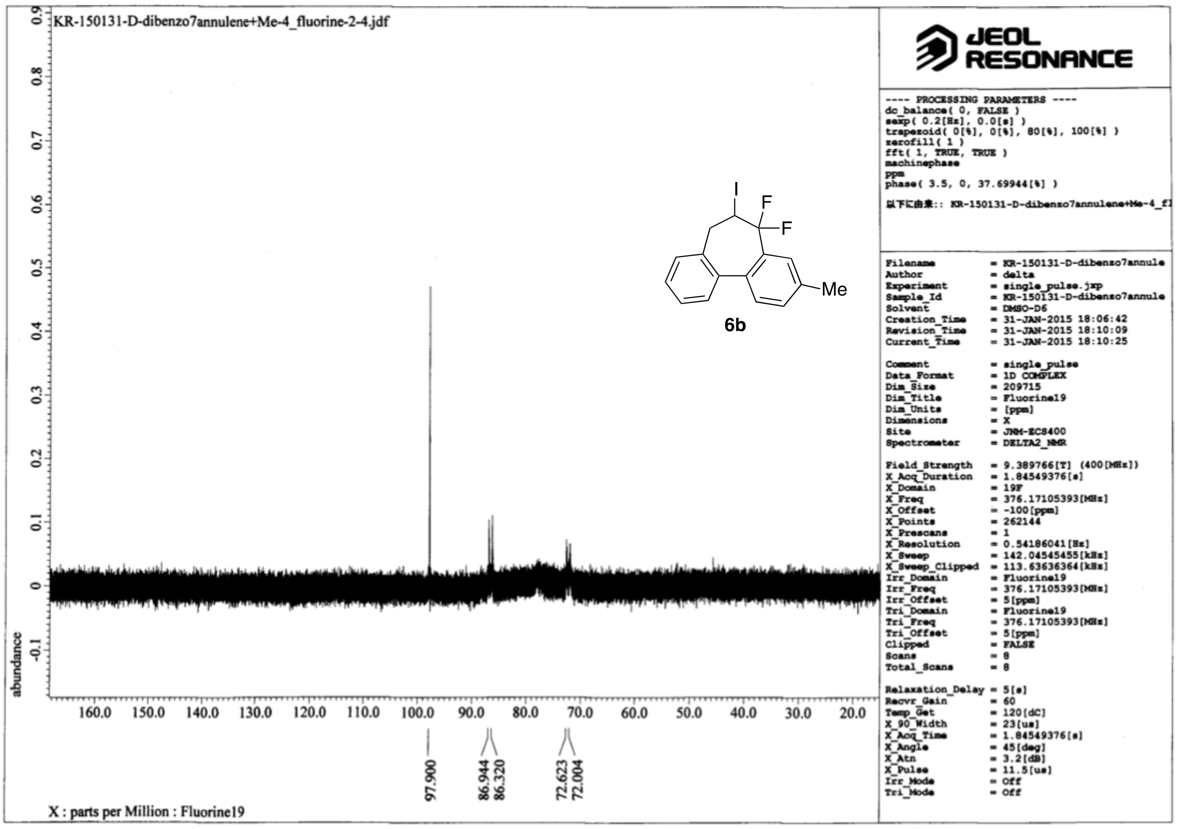
KR-150131-D-dibenzo7annulene+Me-4_fluorine-2-4.jdf
JEOL RESONANCE
I
F
F
Me
6b
abundance
X : parts per Million : Fluorine19
97.900
86.944
86.320
72.623
72.004

**3-Bromo-5,5-difluoro-6-iodo-6,7-dihydro-5*H*-dibenzo[*a*,*c*][7]annulene (6c)**

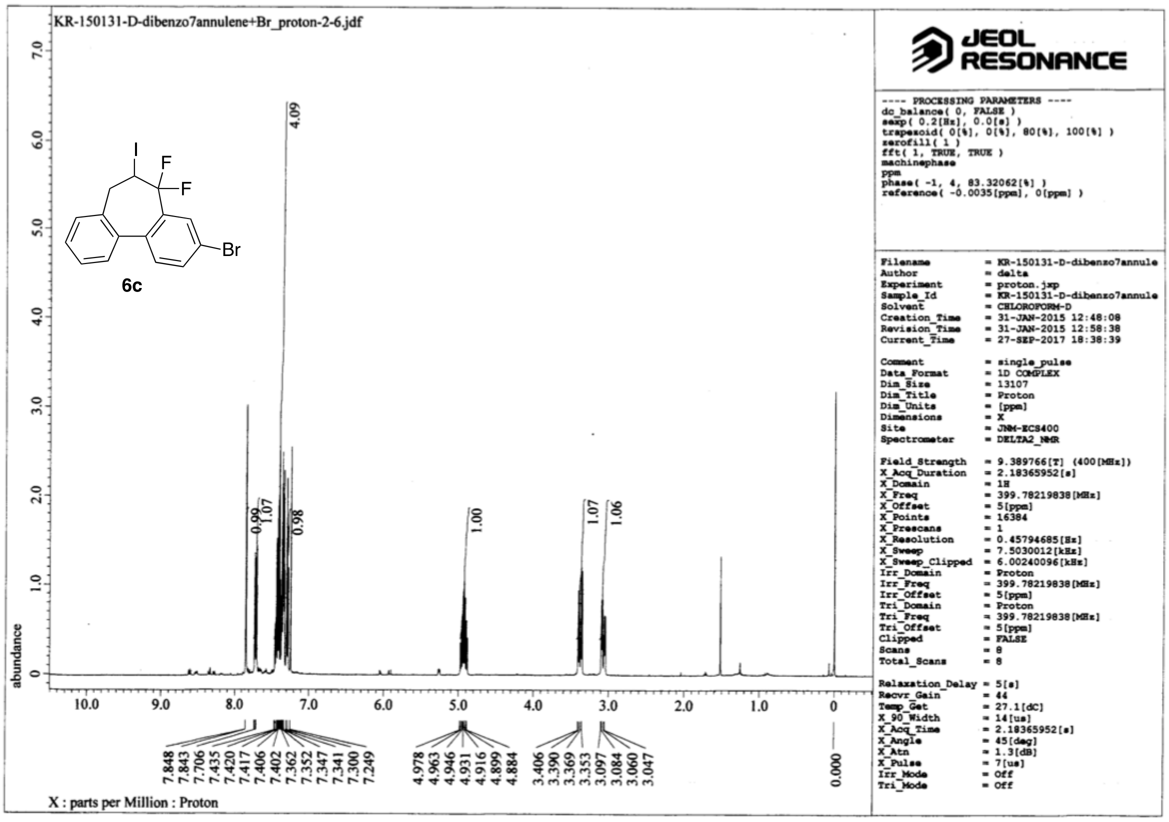

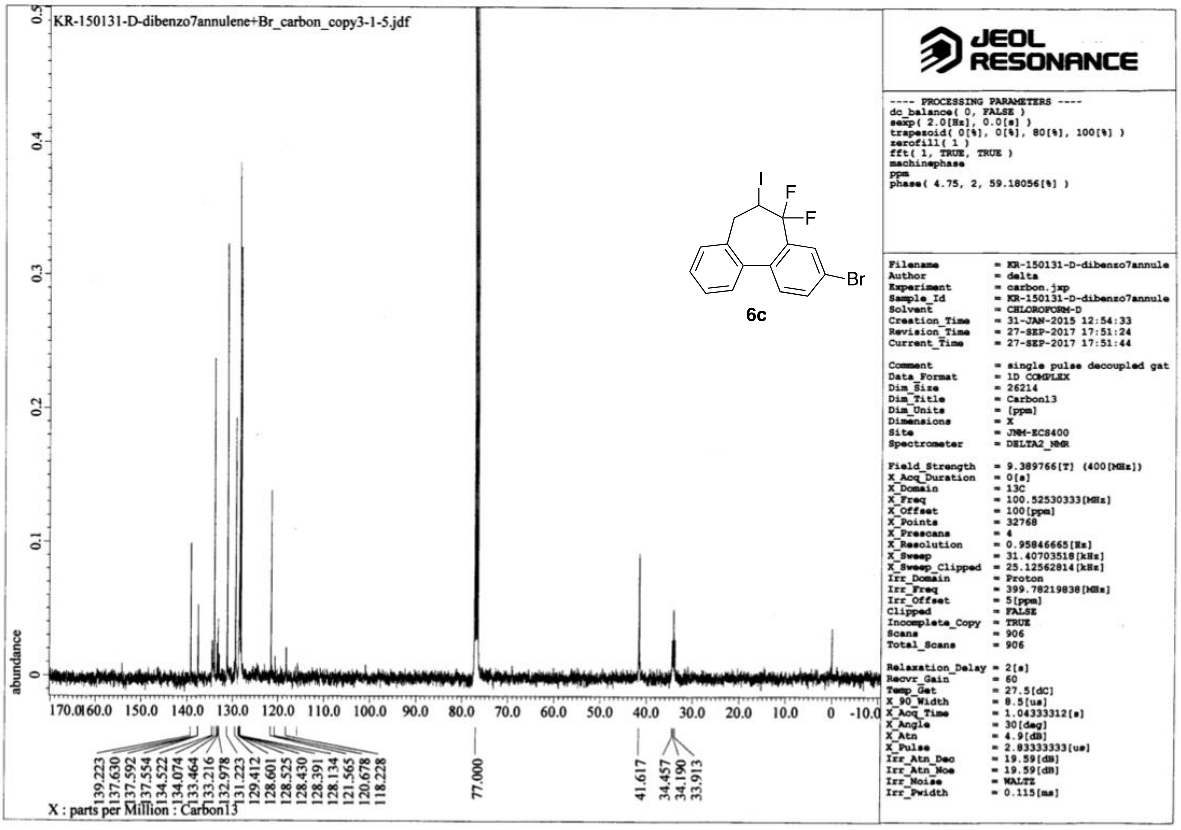

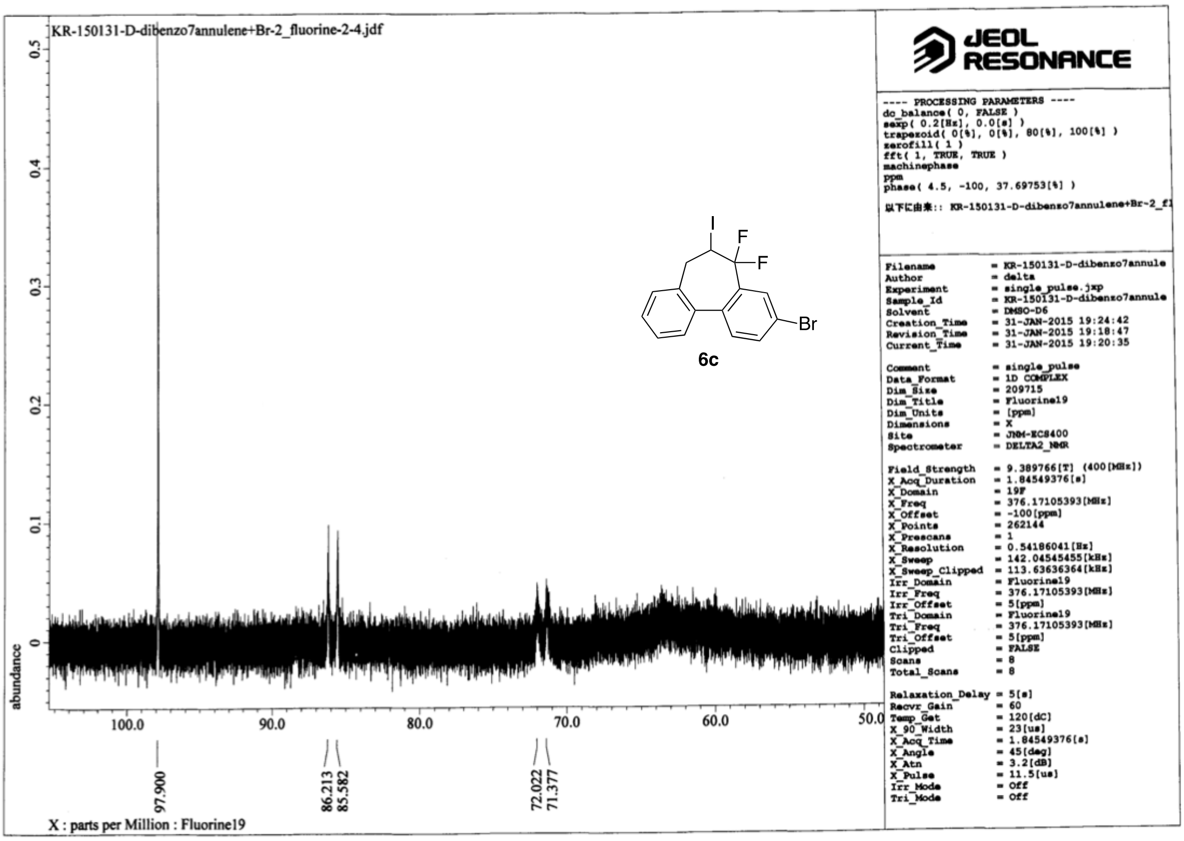


## 5,5-Difluoro-5*H*-dibenzo[*a*,*c*][7]annulene (7a)

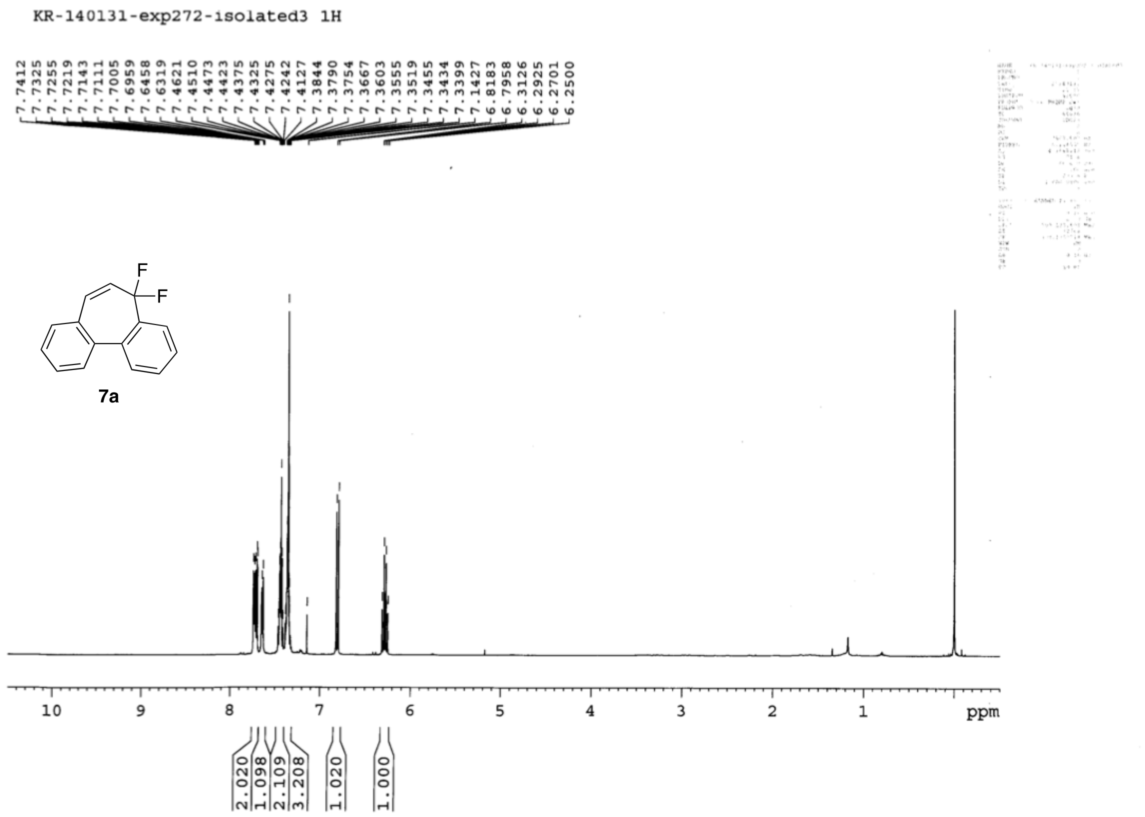

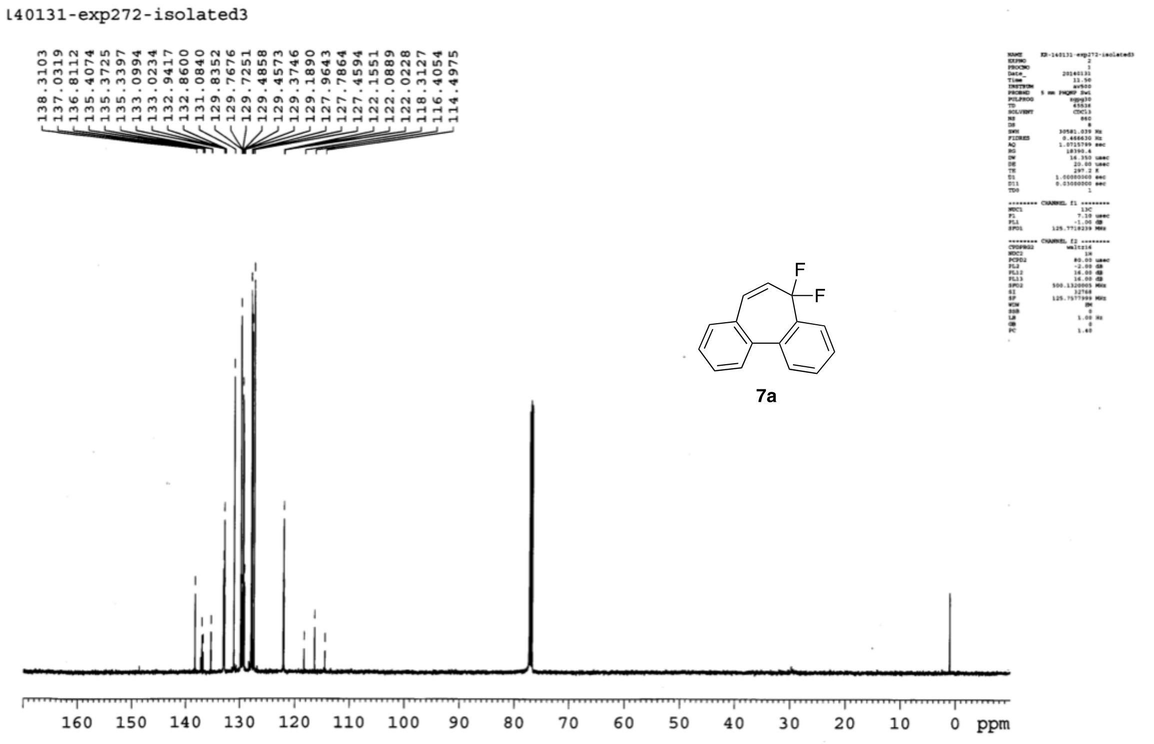

140131-exp272-isolated3
138.3103
137.0319
136.8112
135.4074
135.3725
135.3397
133.0994
133.0234
132.9417
132.8600
131.0840
129.8352
129.7676
129.7251
129.4858
129.4573
129.3746
129.1890
127.9643
127.7864
127.4594
122.1551
122.0889
122.0228
118.3127
116.4054
114.4975
F
F
7a
160 150 140 130 120 110 100 90 80 70 60 50 40 30 20 10 0 ppm


120°C

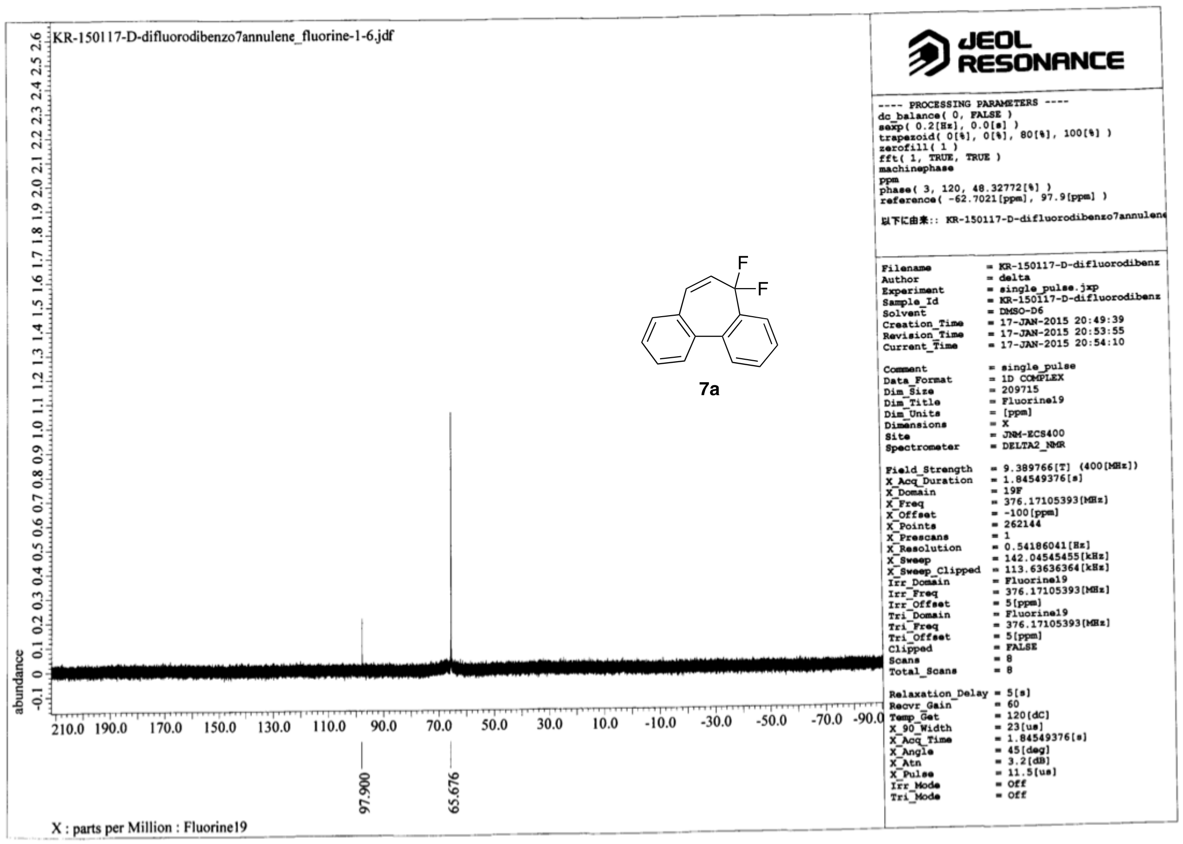

KR-150117-D-difluorodibenzo7annulene_fluorine-1-6.jdf
JEOL RESONANCE
---- PROCESSING PARAMETERS ----
dc_balance( 0, FALSE )
sexp( 0.2[Hz], 0.0[s] )
trapezoid( 0[%], 0[%], 80[%], 100[%] )
zerofill( 1 )
fft( 1, TRUE, TRUE )
machinephase
ppm
phase( 3, 120, 48.32772[%] )
reference( -62.7021[ppm], 97.9[ppm] )
以下に由来:: KR-150117-D-difluorodibenzo7annulene
Filename = KR-150117-D-difluorodibenz
Author = delta
Experiment = single_pulse.jxp
Sample_Id = KR-150117-D-difluorodibenz
Solvent = DMSO-D6
Creation_Time = 17-JAN-2015 20:49:39
Revision_Time = 17-JAN-2015 20:53:55
Current_Time = 17-JAN-2015 20:54:10
Comment = single_pulse
Data_Format = 1D COMPLEX
Dim_Size = 209715
Dim_Title = Fluorine19
Dim_Units = [ppm]
Dimensions = X
Site = JNM-ECS400
Spectrometer = DELTA2_NMR
Field_Strength = 9.389766[T] (400[MHz])
X_Acq_Duration = 1.84549376[s]
X_Domain = 19F
X_Freq = 376.17105393[MHz]
X_Offset = -100[ppm]
X_Points = 262144
X_Prescans = 1
X_Resolution = 0.54186041[Hz]
X_Sweep = 142.04545455[kHz]
X_Sweep_Clipped = 113.63636364[kHz]
Irr_Domain = Fluorine19
Irr_Freq = 376.17105393[MHz]
Irr_Offset = 5[ppm]
Tri_Domain = Fluorine19
Tri_Freq = 376.17105393[MHz]
Tri_Offset = 5[ppm]
Clipped = FALSE
Scans = 8
Total_Scans = 8
Relaxation_Delay = 5[s]
Recvr_Gain = 60
Temp_Get = 120[dC]
X_90_Width = 23[us]
X_Acq_Time = 1.84549376[s]
X_Angle = 45[deg]
X_Atn = 3.2[dB]
X_Pulse = 11.5[us]
Irr_Mode = Off
Tri_Mode = Off
F
F
7a
abundance
-0.1 0 0.1 0.2 0.3 0.4 0.5 0.6 0.7 0.8 0.9 1.0 1.1 1.2 1.3 1.4 1.5 1.6 1.7 1.8 1.9 2.0 2.1 2.2 2.3 2.4 2.5 2.6
210.0 190.0 170.0 150.0 130.0 110.0 90.0 70.0 50.0 30.0 10.0 -10.0 -30.0 -50.0 -70.0 -90.0
97.900
65.676
X : parts per Million : Fluorine19
